# The potential impact of a “curative intervention” for HIV: a modelling study

**DOI:** 10.1186/s41256-019-0107-1

**Published:** 2019-06-12

**Authors:** Leo Beacroft, Timothy B. Hallett

**Affiliations:** MRC Centre for Global Infectious Disease Analysis, Department of Infectious Disease Epidemiology, Imperial College London, London, UK

**Keywords:** HIV epidemiology, Cure, Mathematical modelling, HIV prevention strategies

## Abstract

**Background:**

Efforts to develop an HIV “cure” (i.e., an intervention leading to durable ART-free remission or eradication of HIV infection) have become better resourced and coordinated in recent years. Given, however, the availability of other interventions for prevention and treatment of HIV disease, it is unclear whether, to what extent, and under which circumstances a curative intervention would have an impact in ending the AIDS epidemic and which characteristics of its implementation would be most important. We designed a range of analyses to investigate these unknowns.

**Methods:**

We used a deterministic, compartmental model of HIV infection in South Africa to estimate the impact of a curative intervention. We first examined how its impact would be affected by the state of the epidemic at the time that it is introduced, by the timing and pace of scale-up, and by various targeting strategies. We then investigated the impact of a curative intervention relative to its ability to maintain viral suppression.

**Findings:**

To the extent that other interventions have failed to control the epidemic, i.e., if incidence and AIDS deaths remain high, a curative intervention would result in a larger reduction in incidence. Earlier and faster scale-up allows for greater impact. We also found that a curative intervention would more efficiently reduce transmission if it is prioritised to those not able to obtain or remain on ART and to those aged 15–25 rather than older persons. On the other hand, an intervention that does not maintain viral suppression if the individual is exposed to re-infection could lead to an increase in HIV incidence.

**Conclusions:**

Our findings suggest that a curative intervention for HIV would have the greatest impact if the epidemic is not under control by 2030, particularly if the intervention is targeted to those who are more likely to transmit virus, and if it maintained durable viral suppression, even upon exposure to re-infection. These considerations underscore the need to carefully consider the “target product profiles” for an HIV cure in the context of how and where it would be used, and suggest that such profiles may require revision as the epidemic evolves in the coming years.

**Electronic supplementary material:**

The online version of this article (10.1186/s41256-019-0107-1) contains supplementary material, which is available to authorized users.

## Background

The global response to the HIV epidemic is in a precarious state. Although the scale-up of testing and treatment services has enabled the delivery of antiretroviral therapy (ART) to some 21 million people, or 59% of those living with HIV [1], significant gaps remain. Many people do not start on, or adhere to, ART and this is especially true for young persons. In South Africa, for example, recent data suggest that the percentage of young people (aged 15–24) living with HIV who are on antiretroviral therapy (ART) is only 14.3% compared with 31.2% in the 25–49 age group [2].

Furthermore, HIV incidence remains high in many countries, particularly in certain regions, age groups, and social demographic groups. In South Africa, women aged 15–24 have an annual HIV incidence of 1.51%, three times higher than that found in men (0.49%) and more than 50% higher than the incidence in women aged 15–49 (0.93%) [3]. In sub-Saharan Africa, the number of young people under the age of 25 is projected to increase by over 80% between 2020 and 2060 [4] and this growth could lead to a surge in new infections, elevating the need for ART to even higher (and potentially less sustainable) levels.

These current problems may be mitigated in the future by the development and implementation of different forms of treatment (such as long-acting injectable treatment) and/or prevention technologies (including expanded PrEP or even a vaccine). If not, HIV incidence and AIDS deaths will continue, and alternative approaches may be needed.

An HIV “cure,” i.e., an intervention that could eradicate or suppress the virus in the body in the absence of ART, is increasingly considered a viable target for development. However, it has been uncertain whether or not such an approach should be a priority, and it is not clear what properties it should have. We accordingly constructed a new modelling analysis in order to establish the potential impact of a curative intervention under different scenarios for its properties and its use. We hope that this can aid future research and development by defining some key characteristics of the *‘target product profile’* of the cure.

## Methods

### Epidemic model

Following others [5, 6], we used a deterministic compartmental model of a mature HIV epidemic, calibrated to South Africa. Full details are provided in the online appendix (Additional file 1). Briefly, the population was stratified by sex, male circumcision status, age, and behavioural risk. The HIV-positive population was stratified by CD4 count, ART status, and progression to AIDS. The model includes representations for the expansion of interventions (including ART, male circumcision, and increased condom use) and is calibrated to demographic data on the population age structure as well as HIV prevalence incidence data. The possibility of future interventions (such as long-acting PrEP and a vaccine) is included, drawing on assumptions made by Smith et al. [6].

### Future epidemic scenarios

Uncertainty regarding the future of the epidemic is reflected in three sets of assumptions for the possible future projection (Table 1). In the pessimistic scenario, the epidemic is still not under control by 2030–2050, coverage with ART remains incomplete and other prevention modalities have not become available. In the neutral scenario, the epidemic is reduced by 2030–2050 compared to 2018 levels and coverage of ART is substantially improved although uptake is uneven; however, challenges remain: there is ongoing transmission in key populations and incidence rates among young women remain high as new prevention technologies (e.g., oral PrEP and long-acting PrEP) have not been widely adopted. In the optimistic scenario, the epidemic is reduced substantially by 2030–2050 compared to 2018 levels (near the 90% reduction envisioned the UNAIDS’ Fast Track) due to widespread durable reductions in risk behaviour and the adoption of new prevention technologies, including a partially-effective vaccine; by 2030, ART is provided as a long-acting injectable and uptake is uniformly high across the population.

**Table 1:** Future HIV epidemic scenarios

	Pessimistic scenario	Neutral scenario	Optimistic scenario
Increase in condom coverage (2030 vs 2015)	No increase (85% efficacy)	5 percentage point increase (85% efficacy)	10 percentage point increase (85% efficacy)
ART Coverage	No increase in % PLWHA on ART beyond 2015 (70% efficacy)	By 2030, ART reaches 80–80-80 (80% efficacy)	By 2030, ART reaches 90–90-90 (92% efficacy)
VMMC	Decrease in % adult men circumcised (60% efficacy), reaches 35% by 2050	Increase in % adult men circumcised (60% efficacy), reaches 60% by 2050	Increase in % adult men circumcised (60% efficacy), reaches 70% by 2050
Oral PrEP	None available	Oral PrEP (40% efficacy) reaches 3% coverage by 2030	Oral PrEP (40% efficacy) reaches 10% coverage by 2030
Other Changes	None	None	Long-acting PrEP (75% efficacy) reaches 25% coverage by 2030. Vaccine (70% efficacy) coverage reaches 80% by 2050. Increase in percentage of adult men circumcised beyond 2015 (reaches 70% by 2050)

The assumptions for the future HIV epidemic scenarios investigated in this analysis are given above. Each simulation is repeated for each of the three epidemic scenarios

### Assumptions for baseline scenario

We considered a set of baseline assumptions for a curative intervention:

- The curative intervention becomes available in 2040 and is rolled out across the HIV-infected population (with the exception of those within the 3 months post infection compartment of the model as these individuals are assumed to be not yet diagnosed).
- The curative intervention is given to 10% of the eligible population per year and there is no prioritisation for age or risk groups, and uptake of the intervention is irrespective of ART status.
- There is no risk of relapse and cured individuals can maintain viral suppression even if exposed to re-infection with HIV.

### Scenario analysis

We investigated the effects of varying a number of these assumptions on the potential impact of a curative intervention:

1. To investigate the influence of epidemic context, we examined the impact of an intervention being introduced into each of the three alternative future epidemic projections (Table 1).
2. To investigate the influence of the timing and pace of scale-up, we considered that the intervention could be introduced in 2030, 2040, or 2050, and the pace of scale-up could be 2, 10, or 20% per year.
3. To investigate the influence of an intervention being used by different groups of people, we evaluated scenarios in which it is provided only to persons on ART or only to those not on ART. We also manipulated the relative rate of persons aged 15–24 years old (compared to others) between 1 (‘no prioritisation’) to an 8-fold increase (“age prioritisation”) receiving the intervention. Similarly, in a “risk prioritisation” scenario, only those in the higher risk groups, who, on average, have higher rates of sexual partner change, receive it and those in the low-risk group do not.
4. To investigate the influence of re-infection on the impact of a curative intervention, we compared the baseline assumption to a scenario in which persons benefiting from the intervention have the same risk of infection as those (with the same age, behaviour, and use of interventions) who have never been infected. To investigate the influence of relapse, we compared the baseline assumptions with a scenario in which persons benefiting from the intervention have a risk of relapse (i.e., in which they return to a CD4 cell count between 350 and 500 cells/μl), after an average of 8 or 20 years.

## Results

### The influence of epidemic context

**Fig. 1:**
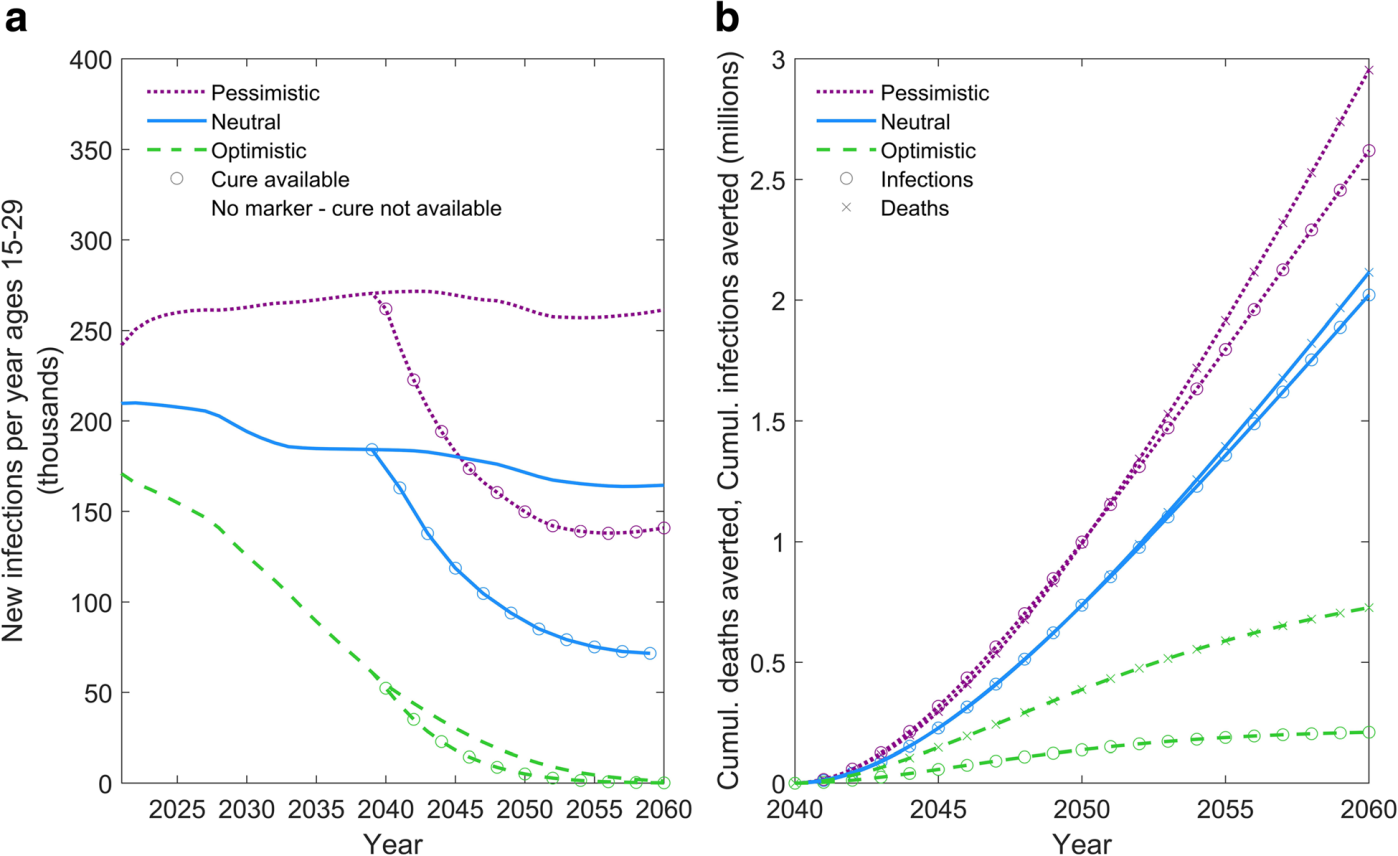
The influence of epidemic context. The impact of the introduction of a curative intervention (under baseline assumptions) scaled-up in the context of either the pessimistic, neutral or optimistic forecasts for the HIV epidemic (Table 1) on (**a**) the number of new infections per year among 15–29 years-old and (**b**) the cumulative infections and deaths averted

Figure 1 shows the impact of introduction of a curative intervention when it is scaled up in each of the three different epidemic contexts (Table 1). In the optimistic scenario, incidence in ages 15–29 is still high in 2030 but is being rapidly reduced as transmission is no longer sustained; by 2050, there are virtually no new infections. Against this background, even the idealised curative intervention has very little impact on new infections, simply because there are few infections to avert. The impact on deaths is more substantial, as the intervention entirely removes the risk of death and it is assumed that, in this scenario, there remains a small risk of HIV-related death for those on ART. In the neutral and pessimistic scenarios, on the other hand, an intervention has substantial impact, reducing the rate of new infections by approximately 50%. Within 20 years of introduction, the idealised curative intervention would have averted up to 2 million infections and 3 million deaths, respectively. The impact is slightly more on deaths than new infections because the intervention directly affects the risk of death for the recipient but only indirectly reduces HIV incidence, and some persons benefitting from the curative intervention would not have passed on the infection even without it.

### The timing and pace of scale-up

**Fig. 2:**
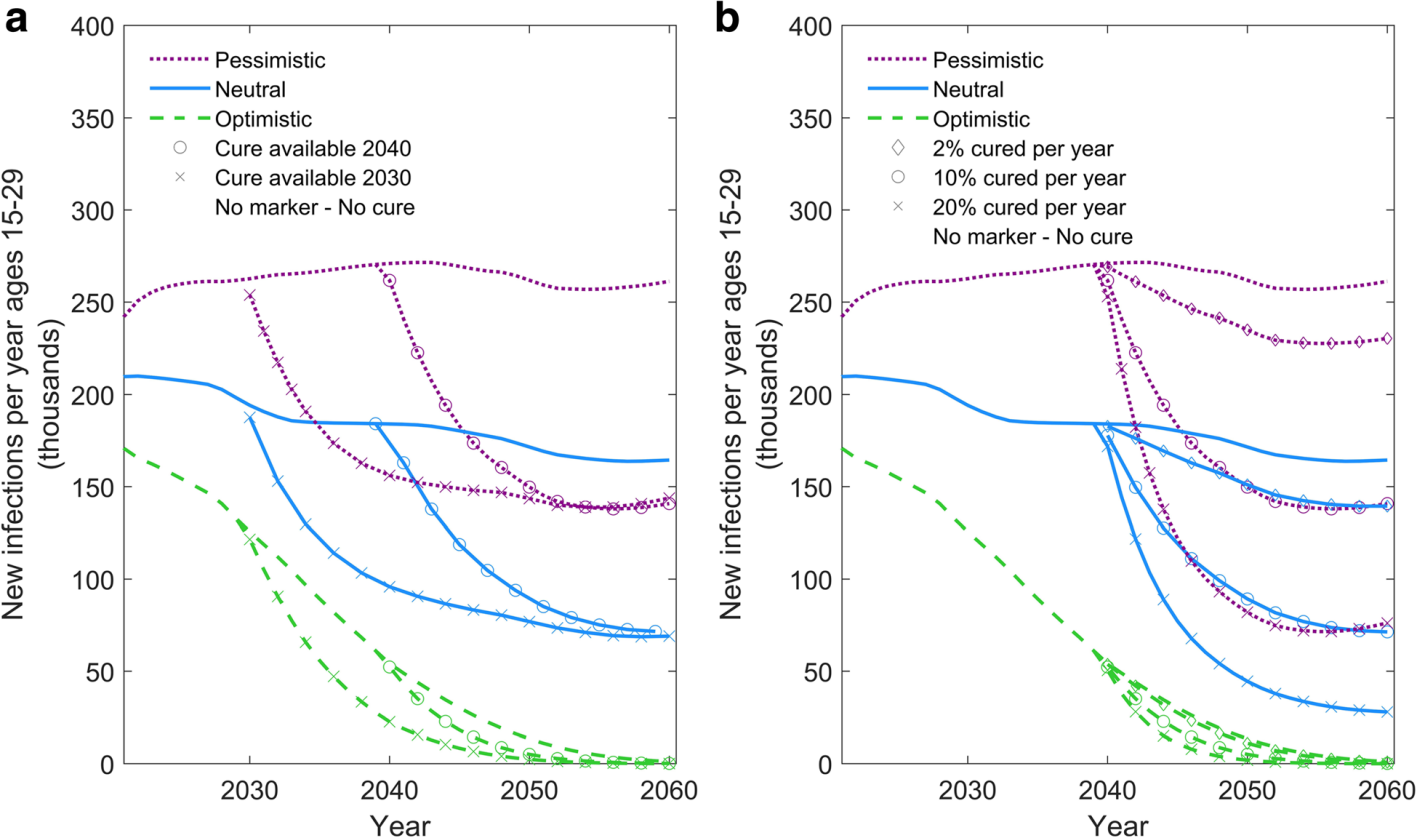
Timing and pace of scale-up. **a** Comparison of two scenarios in which a curative intervention becomes available either in 2040 (baseline) or in 2030. **b** Comparison of different roll-out rates for an intervention in which either 2, 10%, or 20% of eligible HIV-infected individuals are cured per year**.** In both panels, the y-axis shows the number of individuals aged 15–29 newly infected with HIV per year. The green dashed, blue solid, and purple dotted lines represent the optimistic, neutral, and pessimistic epidemic forecasts, respectively (see Table 1). The symbols on the line denote the alternative scenarios for the cure intervention

Figure 2 shows the analysis in which the timing and pace of scale-up are varied. With an earlier introduction of a curative intervention in 2030 compared to 2040, an extra 570,000-1,600,000 infections and 1,300,000 – 2,100,000 AIDS deaths would be averted (depending on the epidemic context) (Fig. 2a). The eventual impact on the youth HIV incidence levels by 2050–2060 is, however, not affected by the timing of scale-up. With a faster scale-up (e.g., 10% or 20% cured per year), the impact of a curative intervention is evident more quickly and also generates a more substantial reduction in eventual levels of HIV incidence and AIDS deaths as more HIV+ individuals benefit before they die. When the scale-up of a curative intervention is as slow as 2% per year, the impact is much reduced at the population level because few benefit from it before dying.

### Efficacy of a curative intervention under different prioritisation scenarios

Table 2 summarises the infections that would be averted per person benefitting from the curative intervention in the context of different scenarios. As expected, the impact of a curative intervention is always more efficient (more infections averted per cure) in the pessimistic epidemic context compared to the optimistic scenario, because there are more infections that can be averted in the pessimistic scenario. Provision of the intervention to those not on ART results in higher efficiency compared to providing the intervention to those already on ART. This is because those not on ART are responsible for more of the on-going transmissions. The loss of efficiency in targeting only those on ART increases under the more optimistic scenarios, as there is then even less risk of transmission arising from persons already on ART. Providing the cure to young persons (15–24 year-olds) gives the highest efficiency for the pessimistic and neutral scenarios. This is because those who are younger are at the beginning of their transmission careers; accordingly, an early intervention can avert a larger amount of transmission risk. Providing the curative intervention to those at higher risk of infection is more efficient than not in the pessimistic epidemic context. In other cases, there is no additional efficiency in targeting the higher risk groups.

**Table 2:** Efficiency of curative intervention under different targeting scenarios

Infections averted per cure (Infections averted per person benefitting from the Curative Intervention)			
	Pessimistic	Neutral	Optimistic
Cure available irrespective of ART status			
No prioritisation	**0.54** (2768/5080)	**0.57** (2143/3731)	**0.18** (212/1169)
Age and risk prioritisation	**0.77** (3097/4033)	**0.64** (1830/2869)	**0.12** (116/930)
Age prioritisation	**1.2** (5302/4449)	**1.1** (3723/3330)	**0.29** (351/1228)
Risk prioritisation	**0.62** (2605/4193)	**0.54** (1573/2931)	**0.1** (95/912)
Cure available only for those On ART			
No prioritisation	**0.22** (684/3181)	**0.28** (856/3069)	**0.14** (142/1046)
Age and risk prioritisation	**0.35** (1222/3444)	**0.39** (1107/2813)	**0.11** (86/810)
Age prioritisation	**0.39** (1467/3737)	**0.54** (1803/3365)	**0.24** (260/1106)
Risk prioritisation	**0.25** (824/3242)	**0.29** (796/2749)	**0.08** (65/787)
Cure available only for those Off ART			
No prioritisation	**0.7** (2179/3125)	**0.87** (1456/1666)	**0.57** (100/175)
Age and risk prioritisation	**0.94** (2807/2973)	**0.93** (1556/1673)	**0.31** (77/251)
Age prioritisation	**1.5** (4598/3045)	**1.7** (3021/1748)	**0.87** (234/270)
Risk prioritisation	**0.74** (2220/3020)	**0.75** (1215/1627)	**0.24** (52/216)

Shown in bold are the number of infections averted per person benefitting from the curative intervention (both counted only in the period 2040–2059). Infections averted are calculated as the difference between the number of infections between 2040 and 2059 in the background scenario in which a curative intervention is not introduced and the number of infections in the comparison scenario between 2040 and 2059 in which one is available. The numbers of infections averted and cures are shown in grey in parentheses. “No prioritisation” means that all age/risk groups have the same access to the curative intervention. “Age-prioritisation” means that persons aged 15–24 years have 8-times the rate of benefitting from the curative intervention as older persons aged 25+ years. “Risk prioritisation” means that only persons in the higher risk group can benefit from the curative interventions

### The properties of the cure

**Fig. 3:**
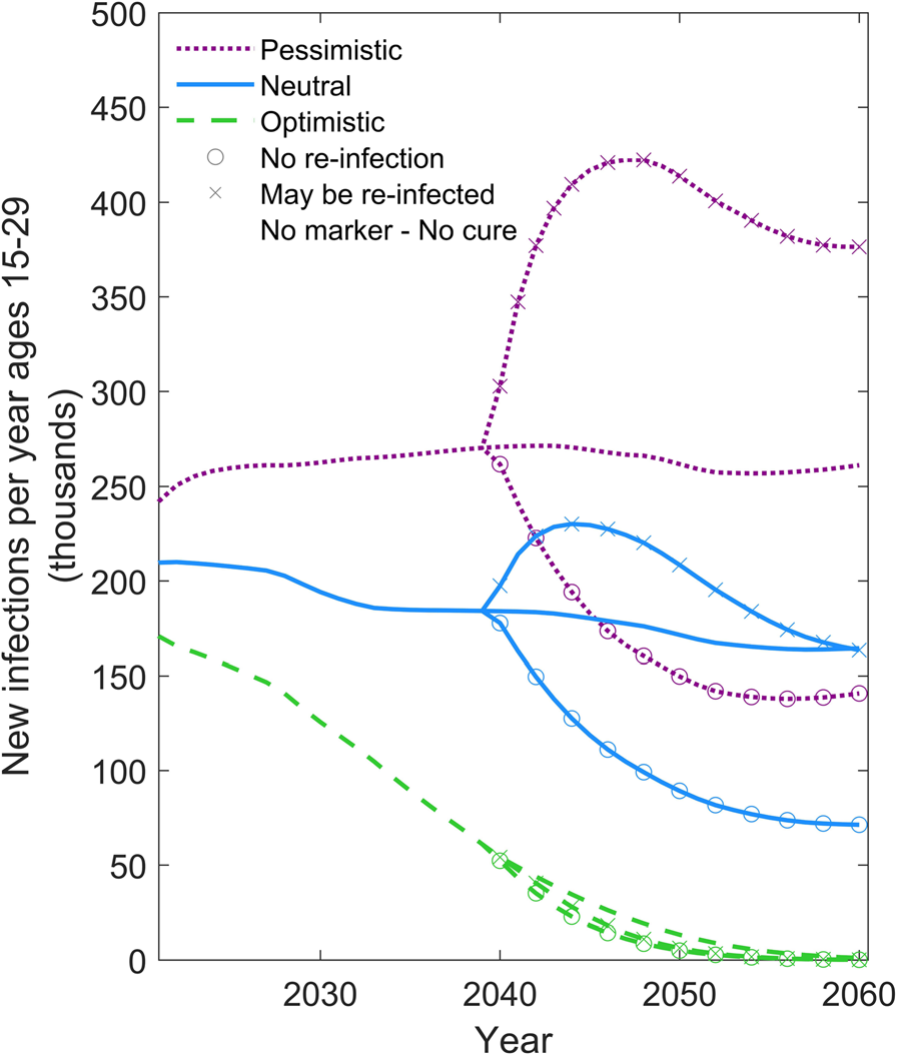
Comparison of a curative intervention that prevents re-infection versus one that allows re-infection. Comparison of scenarios in which a curative intervention either allows for re-infection or prevents subsequent re-infection. The y-axis shows the number of individuals aged 15–29 newly infected with HIV per year. The green dashed, blue solid, and purple dotted lines represent the optimistic, neutral, and pessimistic epidemic forecasts, respectively (see Table 1). The symbols on the line denote the alternative scenarios for the cure intervention

An analysis examining the influence of alternative assumptions about the risk of re-infection following a curative intervention is shown in Fig. 3. A cure that does not provide protection against future re-infection could lead to an increase in HIV incidence in the neutral and pessimistic scenarios because the intervention is effectively increasing the pool of susceptible individuals. This effect is particularly pronounced in the pessimistic scenario as, in this case, the high level of HIV prevalence means that a large number of cures creates a large number of susceptible persons exposed to a high risk of (re-)infection. This effect is muted in the optimistic scenario as the risk of infection is low.

**Fig. 4:**
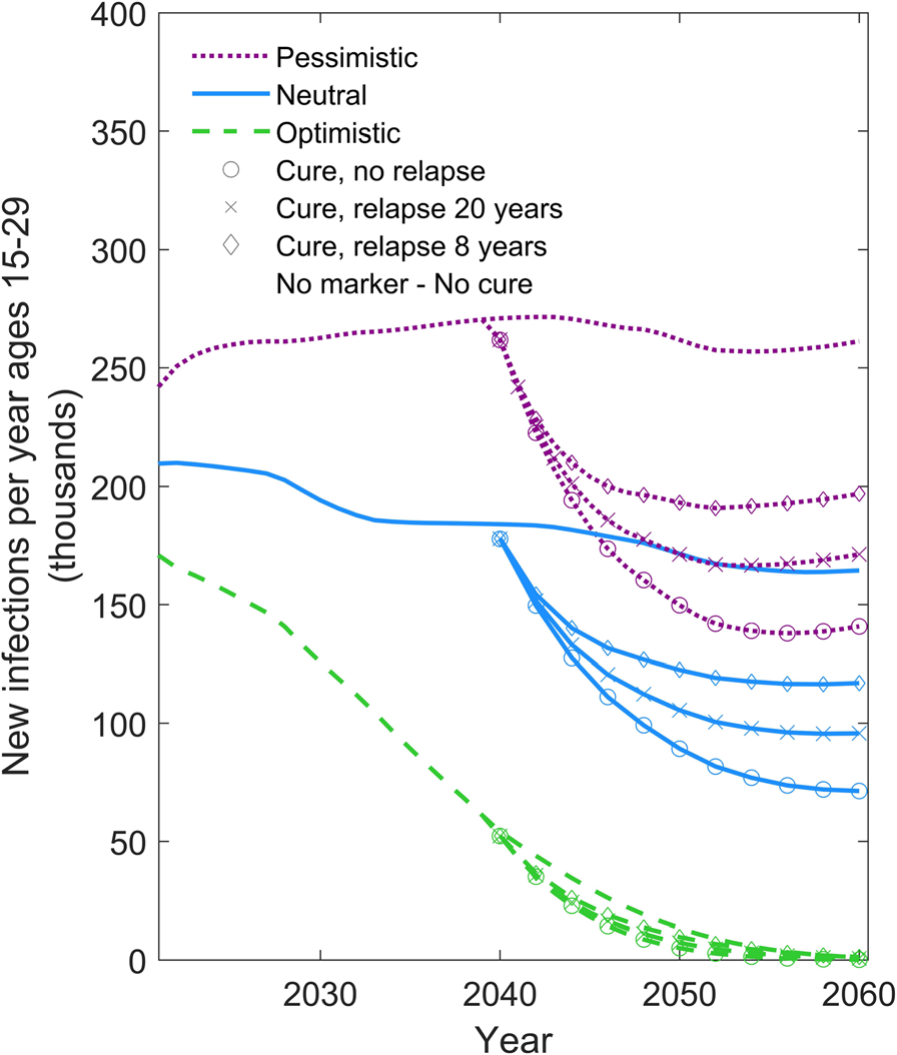
The effect of the possibility for relapse on the impact of a curative intervention. Comparison between scenarios in which relapse is either not possible or occurs after a mean period of 20 years or of 8 years. The y-axis shows the number of individuals aged 15–29 newly infected with HIV per year. The green dashed, blue solid, and purple dotted lines represent the optimistic, neutral, and pessimistic epidemic forecasts, respectively (see Table 1). The symbols on the line denote the alternative scenarios for the cure intervention

An analysis examining the influence of alternative assumptions about the risk of relapse following the cure is shown in Fig. 4. Compared to a curative intervention for which there is no risk of relapse, even a low possibility of a relapse substantially reduces its impact on the epidemic. An intervention with a mean time until relapse of 8 years is projected to have half the impact on reducing new infections by 2060 compared to one with no risk of relapse.

## Discussion

We aimed to investigate the potential impact that an HIV “curative intervention” (i.e., an intervention that leads to durable, ART-free remission or eradication of HIV infection) could have on the HIV epidemic, exploring the impact of varying considerations about its scale-up and intrinsic properties. We found that the impact of a curative intervention is strongly dependent on the state of the epidemic when it is introduced. If the epidemic is well controlled (as in our optimistic scenario), then its impact is low. However, if the epidemic remains uncontrolled, a curative intervention would have a much greater impact. This suggests that investment in research for a cure should be dependent on the projected success of other interventions in controlling the epidemic.

We also found that the sooner a curative intervention is introduced and the more quickly it is scaled-up, the greater the impact it can have. In fact, scaling up a curative intervention 10 years earlier (in 2030 instead of 2040) has a greater influence on the impact of the intervention than other aspects (such as time to relapse). This suggests that scaling up an imperfect intervention sooner may be more impactful that waiting for a perfect curative intervention.

In comparing the impact that the curative intervention has when used among different populations, it was found that the greatest impact per person benefiting from the intervention arises when the intervention is provided to those who are not on ART. This is because the difference in benefits provided by a curative intervention – in terms of both risk of death and risk of onward transmission – would be much greater to someone who is not on ART than in those who are on ART. ‘Cures’ among young persons also tend to have more impact, as the cure will benefit that individual, and the wider population, for a greater proportion of their sexually active lifetime. Whilst it is most likely that a cure would be available to those that are already undergoing treatment, a paramount consideration for the development of a curative intervention should be that its use would be acceptable to those, especially young persons, who may not be willing or able to start and maintain ART.

Finally, we show that the benefit of a curative intervention is effectively negated if it does not continue to suppress viremia upon exposure to re-infection. Indeed, if the epidemic is not controlled – which is the situation in which a curative intervention would be most valuable – there is a risk of causing a rebound in new infections. If there is a risk of relapse from the curative intervention, then this substantially reduces the impact that is generated. Even a long period before a relapse will lead to a high proportion of persons benefiting from the intervention relapsing eventually – especially in the case that the intervention is prioritised to younger persons – and a weakening of impact overall. This will be an important aspect to determine in experiments and trials. Thus, in terms of the required properties for a curative intervention: a low possibility of relapse is important but protection from re-infection is essential. The design of trials to measure these properties will be challenging, however, as short-term follow-up among heavily monitored populations may not accurately evaluate these risks.

In this modelling exercise, we aimed to capture the broad contours of the epidemic and programmatic shifts over time. We did not make assumptions about the details of scale up (e.g., age-specific scale-up rates, sub-group targeting) of particular interventions in the next two decades as this would be speculative. Our scenarios encompass a wide range of trajectories for the epidemic, which believe captures a reasonable range of possibilities, but we note that other epidemic trajectories are possible that may not fit within these bounds. Whilst we accept that there are a number of uncertainties in our projections, our aim at this stage is to gain a basic understanding of the system at a macroscopic level and we believe that the adding an extensive number of uncertainty analyses would be a distraction. One important limitation is that our model is calibrated to South Africa, a generalised epidemic in a high incidence setting. It is possible that a curative intervention may have a different relative impact in a lower incidence country or an epidemic that is more concentrated among key populations.

A curative intervention may have further benefits that are not captured here. In particular, curing large proportions of the population could reduce a number of non-communicable diseases that are associated with long-term HIV [7]. Also, the management of comorbidities may become easier, as ART and HIV currently create a complex of contra-indications due to drug-drug interactions [8].

Previous modelling analyses have helped to provide insights into some questions related to the impact of a cure for HIV disease. Phillips et al. [9] focussed on an intervention that becomes available in 2022 and is given only to those on ART. The authors investigated the cost-effectiveness of a curative intervention under different rollout scenarios and, in contrast to the results presented here, found that uncertainty in future HIV incidence and prevalence have a limited impact on the results. This difference may be due to an earlier introduction of the intervention: in our analyses, the cure is introduced in 2040 or 2030 and, hence, there has been more time for the divergence of different epidemic trajectories.

Dimitrov et al. [10] investigated the impact of a curative intervention under two availability scenarios, one in which it is only available to those on suppressive ART and another in which it is made available irrespective of ART status. The authors found that HIV incidence would not be reduced unless the intervention was available to ART-naïve individuals. Similarly, we report here that the efficiency of a curative intervention can be improved by targeting those not on ART. However, we find that incidence can be reduced without targeting specifically to those off ART. The difference in findings may be due to differences in assumptions for the proportion of the HIV-infected population on ART. Our assumption is that between 50 and 87% of the HIV-infected population are on ART when the cure becomes available in 2040, whereas Dimitrov et al. assumed 20% of the infected population are on ART.

Curative interventions are available for a number of other sexually transmitted diseases (STDs). Antibiotics have been available for many decades, yet bacterial STDs are still prevalent. This suggests that eradication may require not only an available cure, but also other interventions, such as a rigorous testing programme and large-scale uptake of preventative measures. Notably, it is the optimistic scenario, in which it is assumed that there is continued scale up of ART and male circumcision, as well as the introduction of new interventions, in which HIV comes closest to reaching zero new infections. One implication of this observation is that scale-up of other interventions remains important for reducing HIV incidence and prevalence. It is possible that the focus of funders, health-care providers and patients might immediately shift towards a curative intervention, were it to become available. However, given the time that would be required for the curative intervention to be scaled up, waning interest in ART, PrEP and other interventions could mitigate the impact of the cure. This suggests that even if it were known that a cure will be available in the future, it nevertheless remains important that there is continued focus on existing forms of treatment and prevention. Given the interplay between the impact of a cure and the effectiveness of other forms of epidemic control, a final consideration is that, in the scaling up of a cure, situations should be avoided whereby persons may wish to cease taking ART in order to increase their chance of receiving a cure, or persons cease using other forms of HIV prevention as they perceive that the threat of HIV is diminished, as each of these would ultimately undermine the chance of seeing further reductions in HIV infections and AIDS deaths.

In sum, our analysis suggests several important features worthy of consideration when constructing a target product profile for an HIV “cure:” first, the intervention would best be one that can be adopted by those not able to access or to stay on ART; secondly, it should continue to suppress viraemia even after exposure to re-infection; and, finally, the risk of relapse must be low. There are a number of ways in which a curative intervention could be developed [11], and one or more of these avenues may warrant significant further investment and development. Given the extensive difficulties associated with each possible pathway, it is important to consider the target product profile of an optimal curative intervention (which may vary as the HIV epidemic matures) such that it can have the greatest possible impact. Should a curative intervention of this type be introduced into resource-limited parts of the world where transmission rates remain high and ART coverage low, it is likely that it will provide health benefits to those who are treated, reduce the risk of transmission to those who are uninfected, and free up resources to better diagnose and treat those who are infected.

## Additional files

Additional file 1: Supplemenatry Methods Information. (DOCX 2752 kb)

## Data Availability

The datasets supporting the conclusions of this article are included within the article and its additional files.
