# Correction to: The potential impact of a “curative intervention” for HIV: a modelling study

**DOI:** 10.1186/s41256-020-00173-0

**Published:** 2020-10-21

**Authors:** Leo Beacroft, Timothy B. Hallett

**Affiliations:** Department of Infectious Disease Epidemiology, Imperial College London, MRC Centre for Global Infectious Disease Analysis, London, UK

**Correction to: Glob Health Res Policy 4, 18 (2019)**

**https://doi.org/10.1186/s41256-019-0107-1**

Following publication of the original article [1], the authors identified an error in Fig. 3. The correct figure is given below.

**Fig. 3:**
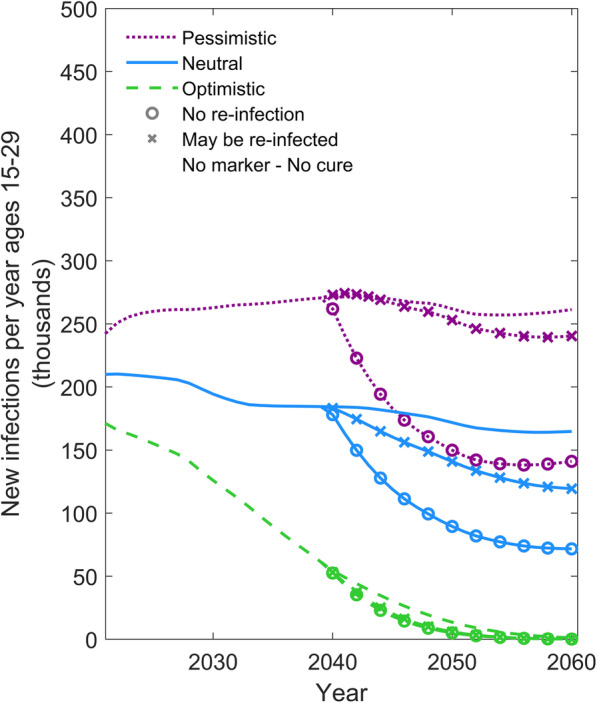
Comparison of a curative intervention that prevents re-infection versus one that allows re-infection. Comparison of scenarios in which a curative intervention either allows for re-infection or prevents subsequent re-infection. The y-axis shows the number of individuals aged 15–29 newly infected with HIV per year. The green dashed, blue solid, and purple dotted lines represent the optimistic, neutral, and pessimistic epidemic forecasts, respectively (see Table 1). The symbols on the line denote the alternative scenarios for the cure intervention

As the result, some of the main text needs to be adjusted:

## Paragraph 1 of “The properties of the cure”

An analysis examining the influence of alternative assumptions about the risk of re-infection following a curative intervention is shown in Fig. 3. A cure that does not provide protection against future re-infection could lead to a small increase in HIV incidence in the years immediately following the introduction of the cure because the intervention is effectively increasing the pool of susceptible individuals. This occurs in the pessimistic scenario, due to the high level of HIV prevalence, which means that a larger number of people are cured, which in turn creates a large number of susceptible persons exposed to a high risk of (re-)infection. In the neutral scenario, the rate of new infections in 15–29 year olds decreases following the introduction of the cure, yet the magnitude of the decrease in the infection rate is much smaller than the scenario in which the cure prevents re-infection. The effect of re-infection is muted in the optimistic scenario as the overall level of HIV incidence is low.

## Paragraph 2 in Discussion

We also found that the sooner a curative intervention is introduced and the more quickly it is scaled-up, the greater the impact it can have. In fact, scaling up a curative intervention 10 years earlier (in 2030 instead of 2040) has a greater influence on the impact of the intervention than other aspects (such as time to relapse). This suggests that scaling up an imperfect intervention sooner may be more impactful than waiting for a perfect curative intervention.

## Paragraph 4 in Discussion

Finally, we show that the benefit of a curative intervention is reduced if it does not continue to suppress viremia upon exposure to re-infection. Indeed, if the epidemic is not controlled – which is the situation in which a curative intervention would be most valuable – there is a risk of causing a rebound in new infections. However, in our long term projections, we find an overall reduction in new infections under all epidemic scenarios considered here. If there is a risk of relapse from the curative intervention, then this substantially reduces the impact that is generated. Even a long period before a relapse will lead to a high proportion of persons benefiting from the intervention relapsing eventually – especially in the case that the intervention is prioritised to younger persons – and a weakening of impact overall. This will be an important aspect to determine in experiments and trials. Thus, in terms of the required properties for a curative intervention: a low possibility of relapse is important as is some degree of protection from re-infection. The design of trials to measure these properties will be challenging, however, as short-term follow-up among heavily monitored populations may not accurately evaluate these risks.
