# Supplementary material for: The potential impact of a “curative intervention” for HIV: a modelling study
Source: Glob Health Res Policy. 2019 Jun 12;4:2. doi: 10.1186/s41256-019-0107-1 (PMC6567561; doi:10.1186/s41256-019-0107-1)
Supplement: Supplementary file 1 — Supplemenatry Methods Information. (DOCX 2752 kb) [file 41256_2019_107_MOESM1_ESM.docx]

**The Potential Impact of a “Curative Intervention” for HIV:**

**A Modelling Study**

**Supporting Information**

South Africa Epidemic Model

# 2.1 Model overview

Based on previous model developed by Cremin and co-authors [1,2], we adapted a deterministic compartmental model defined by a set of ordinary differential equations. It is designed to represent heterosexual HIV transmission at the population level in South Africa, a mature, generalised HIV epidemic. Our aim is to estimate how the future introduction of curative interventions for HIV could impact the epidemic. By comparing different curative interventions, this analysis may aid future research by informing upon the *‘target product profile’* of a curative intervention for HIV.

The model population is divided into compartments that are distinguished by sex, circumcision status (if male), age, infection stage, sexual behaviour, and contraceptive use, with events (e.g. HIV infection, death, ART initiation etc.) represented as movement between these compartments [3–5]. Heterogeneity in sexual behaviour is incorporated in the model by stratifying men and women into three risk groups according to their average effective partnership formation rate.

A full description of the model structure, parameter values used, and calibration is provided under the following sections: 2.2 Natural History of HIV infection, 2.3 Demography, 2.4 HIV transmission and sexual mixing, 2.5 Male circumcision, 2.6 Antiretroviral treatment, 2.7 Contraception, 2.8 Model calibration.

# 2.2 Natural history of HIV infection

A flow diagram for the natural history of HIV infection and treatment cascade is shown in Figure S1.


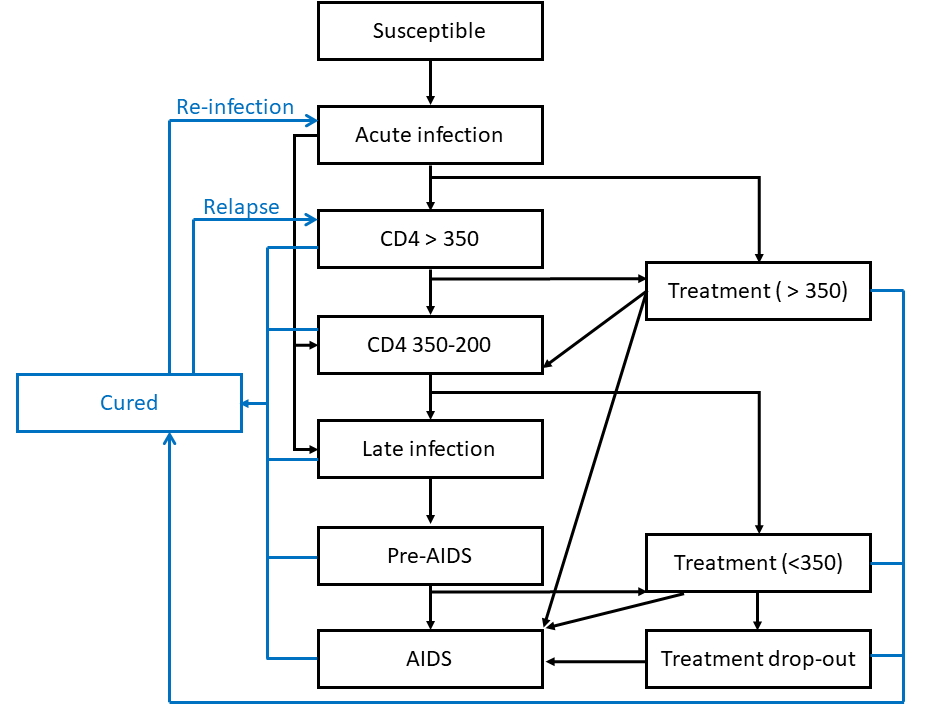


*Figure S1: Natural history of HIV infection and ART initiation as represented in the model*

*Movement between compartments is indicated by arrows. Shown in blue are the effects of the curative intervention. Some movement between compartments (e.g. re-infection and relapse) occurs only for specific sub-analyses.*

The model is specified by the following ordinary differential equations:

The equations describing susceptible individuals are:

$\frac{dX_{l,k,1}^{0,p}}{dt}=\mu N\Psi_{\left( l,k,p,1 \right)}-\left( \lambda_{l,k,1}^{p}+\mu_{k,1} \right)X_{l,k,1}^{0,p}+f\left( 1 \right)+g(k)+ q(p)$ for a=1

$\frac{dX_{l,k,a}^{0,p}}{dt}=-\left( \lambda_{l,k,A}^{p}+\mu_{k,a} \right)X_{l,k,a}^{0,p}+f\left( a \right)+g(k)+ q(p)$ for a≠1

(1)

The equations describing HIV infected individuals are:

$$\frac{dX_{l,k,a}^{1,p}}{dt}=\lambda_{l,k,A}^{p}X_{l,k,a}^{0,p}-\left( {\left( \gamma1+\gamma2+\gamma3 \right)\sigma}_{1}+\mu_{k,a} \right)X_{l,k,a}^{1,p}+f\left( a \right) + g\left( k \right)+ q\left( p \right)$$

$$\frac{dX_{l,k,a}^{2,p}}{dt}=\left( 1-\phi_{1} \right)\sigma_{1}{\gamma1X}_{l,k,a}^{1,p}+ \varsigma X_{l,k,a}^{10,p}+\zeta_{E}X_{l,k,a}^{7,p}-\left( \sigma_{2}+\mu_{k,a}+ \nu_{2} \right)X_{l,k,a}^{2,p}+f\left( a \right)\ldots+g\left( k \right)+q\left( p \right)$$

$$\frac{dX_{l,k,a}^{3,p}}{dt}=\left( 1-\phi_{2} \right)\sigma_{2}\gamma2X_{l,k,a}^{2,p}-\left( \sigma_{3}+\mu_{k,a}+\nu_{3} \right)X_{l,k,a}^{3,p}+f\left( a \right)+ g(k)+ q(p)$$

$$\frac{dX_{l,k,a}^{4,p}}{dt}=\left( 1-\phi_{3} \right)\sigma_{3}\gamma3X_{l,k,a}^{3,p}-\left( \sigma_{4}+\mu_{k,a}+\nu_{4} \right)X_{l,k,a}^{4,p}+f\left( a \right)+ g(k)+ q\left( p \right)$$

$$\frac{dX_{l,k,a}^{5,p}}{dt}=\sigma_{4}X_{l,k,a}^{4,p}-\left( \sigma_{5}+\mu_{k,a}+\nu_{5} \right)X_{l,k,a}^{5,p}+f\left( a \right)+ g(k)+ q(p)$$

$$\frac{dX_{l,k,a}^{6,p}}{dt}=\left( 1-\phi_{4} \right)\sigma_{5}X_{l,k,a}^{5,p}+\omega_{E}X_{l,k,a}^{7,p}+\omega_{L}X_{l,k,a}^{8,p}+\tau_{D}X_{l,k,a}^{9,p}-\left( \Omega+\mu_{k,a}+\nu_{6} \right)X_{l,k,a}^{6,p}+f\left( a \right)\ldots+ g\left( k \right)+ q\left( p \right)$$

$$\frac{dX_{l,k,a}^{7,p}}{dt}={\phi_{3}\sigma}_{3}X_{l,k,a}^{3,p}+{\phi_{4}\sigma}_{5}X_{l,k,a}^{5,p}-\left( \omega_{L}+\zeta_{L}+\mu_{k,a}+\nu_{7} \right)X_{l,k,a}^{7,p}+f\left( a \right)+g(k)+q(p)$$

$$\frac{dX_{l,k,a}^{8,p}}{dt}={\phi_{1}\sigma}_{1}\gamma1X_{l,k,a}^{1,p}+{\phi_{2}\sigma}_{2}X_{l,k,a}^{2,p}-\left( \omega_{E}+\zeta_{E}+\mu_{k,a}+\nu_{8} \right)X_{l,k,a}^{8,p}+f\left( a \right)+g\left( k \right)\ldots. +q(p)$$

$\frac{dX_{l,k,a}^{9,p}}{dt}=\zeta_{L}X_{l,k,a}^{8,p}-\left( \tau_{D}+\mu_{k,a}+\nu_{9} \right)X_{l,k,a}^{9,p}+f\left( a \right)+ g(k)+ q(p)$

$\frac{dX_{l,k,a}^{10,p}}{dt}=\sum_{i=2}^{9} {\nu_{i}X}_{l,k,a}^{i,p} -({\pi\lambda}_{l,k,A}^{p}+ \mu_{k,a}+ \varsigma)X_{l,k,a}^{10,p}$ $+f\left( a \right)+ g(k)+ q(p)$

(2)

For a given stage of HIV infection, hazards of progression to the next stage are given by the rates σ _(1,2,3,4,5)_. The late infection stage is defined by the mean time between when CD4 count falls below 200 cells/μl and viremic rebound 19 months, on average, before death. The pre-AIDS stage characterizes the 9 month period of heightened infectiousness before AIDS, which represents a 10 month period of no transmission risk [6].

Mortality in the AIDS stage is denoted by the parameter Ω. An important limitation of this model is that AIDS-related mortality only applies to this final AIDS stage. However, in reality some infected individuals may die of AIDS-related illnesses at higher CD4 counts.

Several representations of ART initiation are possible in the model; ART can be initiated following acute infection, when an individual’s CD4 count drops below 350, 200, or 100 cells per microliter. The proportion of individuals initiating ART following acute infection, at CD4 <350 cells/μl, CD4 <200 cells/μl and CD4 <100 cells/μl are controlled by the parameters *ϕ_1,_ ϕ_2_*, *ϕ_3,_* and *ϕ_4,_* respectively*_._*  ART initiation at low CD4 counts (< 100 cells/μl) is used to represent the initial pattern of ART initiation (i.e. for urgent clinical need), when ART was first introduced in South Africa.

ART is assumed to extend the survival of treated individuals (the increase in life expectancy depends on whether ART is initiated ‘≥350 cells/μl’ or ‘<350 cells/μl’) while reducing infectiousness [7,8] . Individuals initiating ART ≥350 cells/μl or <350 cells/μl are assumed to survive on average $\frac{1}{\omega_{E}}$ or $\frac{1}{\omega_{L}}$ years before progressing to AIDS, respectively. Drop outs from treatment initiated ≥350 cells/μl return to having a CD4 ≥350 cells/μl and progress through infection. Drop outs from treatment when initiated <350 cells/μl’ progress to AIDS after a period of slightly heightened infectiousness represented by the ‘Treatment drop-out’ compartment in Figure S1.

A curative intervention is modelled by moving individuals from all infectious stages, except the acute infection stage in which HIV positive individuals are assumed to be undiagnosed, to a cured category. Individuals move from infectious classes *X*^i^ to the cured class *X^10^* at a rate *ν_i_.*

Individuals in the cured class are assumed to be immune to reinfection in the baseline assumption, but a sub-analysis investigates the impact of allowing reinfection at a rate ${\pi\lambda}_{l,k,A}^{p}$ where $\lambda_{l,k,A}^{p}$ is the force of infection and $\pi$ is a coefficient, defined as 0 for the baseline assumption and 1 for the sub-analysis in which reinfection is possible. Individuals relapse from the cured state to the infected state with a CD4 cell count > 350 cells µl^-1^ at a rate $\varsigma$. For the baseline assumption, in which relapse does not occur $\varsigma=0$, but for the sub-analysis in which relapse is possible, $\varsigma=\frac{1}{8}$ years^-1^ or$\varsigma=$ $\frac{1}{20}$ years^-1^.

| **Parameter** | **Symbol** | **Value** | **Source** |
| --- | --- | --- | --- |
| Mean duration of acute infection | 1/σ_1_ | 0.25 years | [6] |
| Mean duration from the end of acute infection to CD4 350 cells/μl | 1/σ_2_ | 9.25 years |  |
| Mean duration from CD4 350 cells/μl to CD4 200 cells/μl | 1/σ_3_ | 3.54 years |  |
| Mean duration from CD4<200 cells/μl to viremic rebound | 1/σ_4_ | 1.12 years |  |
| Mean duration of viremic rebound before AIDS | 1/σ_5_ | 0.75 years |  |
| AIDS mortality rate | Ω | 1/0.833 (10 month period before death) |  |
| Proportion individuals in the acute stage of HIV infection moving to CD4> 350 cells/μl | $\gamma1$ | 0.81  (0.58+0.23) | [9] |
| Proportion of individuals in the acute stage of HIV infection moving to CD4 200-350 cells/μl | $\gamma2$ | 0.16 |  |
| Proportion of individuals in the acute stage of HIV infection moving to CD4<200 cells/μl | $\gamma3$ | 0.03 |  |
| Mean time to relapse after cure | $1/\varsigma$ | 8 years, 20 years or $\varsigma=0$ (no relapse |  |
| Reinfection coefficient | π | 0 (no reinfection) or 1 (reinfection possible) |  |

***Table S1: Natural history of infection parameters***

# Demography

The model is stratified by one-tenth of a single year of age from birth to 100 years. Ageing of individuals is represented by:

$$X_{l,k,1}^{0,p}= X_{l,1,a}^{s,p}b_{a} for a=1$$

$$X_{l,k,a}^{s,p}=X_{l,k,a-1}^{s,p} for a>1$$

(3)

$\Psi_{\left( l,k,p,a \right)}$ is the matrix of population distribution in the year the epidemic starts (*t_0_*) over each *l, k, a* stratum and it is defined in terms of: (i) $\varphi_{f(l)}$ and $\varphi_{m(l)}$which are the proportion of females and males respectively in each risk activity group; and (ii) *f_a_* which is the proportion of the population in each year of age, with $\sum_{a=1}^{100} f_{a}=1$. The parameter *f_cm_* gives the fraction of males who are circumcised.

The total number in the population (N) and $\Psi_{\left( l,k,p,a \right)}$ are given by:

$$N=\sum_{l=1}^{3} \sum_{k=1}^{3} \sum_{p=1}^{7} \sum_{a=1}^{100} \left( X_{l,k,a}^{0,p}+X_{l,k,a}^{1,p}+X_{l,k,a}^{2,p}+X_{l,k,a}^{3,p}+X_{l,k,a}^{4,p}+X_{l,k,a}^{5,p}+X_{l,k,a}^{6,p}+X_{l,k,a}^{7,p}+X_{l,k,a}^{8,p}+X_{l,k,a}^{9,p} \right)$$

(4)

$$\Psi_{\left( l,k,p,a \right)}=\left\{ \begin{aligned} \frac{1}{2}\varphi_{f(l)}f_{p} f_{a} any l; k=1;any p; any a; \\ \frac{1}{2}\varphi_{m(l)}f_{a}\left( 1-f_{cm} \right) any l; k=2; p=1; any a; \\ \frac{1}{2}\varphi_{m(l)}f_{a}f_{cm} any l; k=3;p=1; any a; \end{aligned} \right.$$

(5)

Individuals enter the population as susceptible at birth (i.e., age zero), the distribution of whom is defined by the population distribution matrix over each *l, k, and p* stratum ($\Psi_{\left( l,k,p,1 \right)}$), given by:

$$\Psi_{\left( l,k,1,1 \right)}=\left\{ \begin{aligned} \frac{1}{2}\varphi_{f\left( l \right)} any l; k=1;p=1 (no method); a=1 (0 years); \\ \frac{1}{2}\varphi_{m\left( l \right)}\left( 1-f_{cm} \right) any l; k=2;p=1 (no method); a=1 (0 years); \\ \frac{1}{2}\varphi_{m\left( l \right)}f_{cm} any l; k=3; p=1 (no method); a=1 (0 years); \end{aligned} \right.$$

(6)

Age-specific fertility rates (*b_a,t_*) and age- and sex-specific non-AIDS mortality rates (*μ_k,a,t_*) are taken from the ASSA 2008 model and are updated each year from 1985 to 2025 as estimated by that model [10]. AIDS-related mortality is modeled explicitly (Table S1). The South African population distribution by age in 1985 (*f _a_*) is taken from the same source [10]. The fraction of the female population using each contraceptive method ($f_{p})$ is based on the South African National HIV Prevalence, Incidence and Behaviour Survey, 2012 [11].

# 2.4 HIV transmission and sexual mixing

##### Force of infection

The force of infection is the per capita rate at which susceptible individuals acquire infection. Following previous work [1], the per capita force of infection $\lambda_{l,k,A}^{p}$ is the force of infection experienced by individuals of each contraceptive group, risk group, sex, circumcision status (if male), and five year age group from the infected population of the opposite sex at a given time. Characteristics of an individual (p, l, k, and A (where A is five-year age group)) are distinguished from those of their sexual partners by means of a prime (i.e. p’, l’, k’ and A’). The force of infection is calculated by five-year age group and then applied to each single year of age in that group.

The force of infection depends on the pattern of partnership formation between different risk and five-year age groups and on the probability of transmission per partnership as well as DMPA usage by female partners, and is defined as:

For women:

$$\lambda_{l,1,A}^{p}=\sum_{l'} \sum_{k'} \sum_{p'} \sum_{A'} \sum_{s'} \left[ C_{g,A,l}\rho_{g,A,l,A^{'},l^{'}}\left( \frac{X_{l^{'},k^{'},A^{'}}^{s^{'}, p^{'}}}{\sum_{k^{'}=2}^{3} \sum_{p'} \sum_{s'} X_{l^{'},k^{'},A^{'}}^{s^{'},p^{'}}} \right)Z_{l,1,p,s^{'},l^{'},k^{'},p^{'}} \right]$$

For men:

$$\lambda_{l,k,A}^{p}=\sum_{l'} \sum_{p'} \sum_{A'} \sum_{s'} \left[ C_{g,A,l}\rho_{g,A,l,A^{'},l^{'}}\left( \frac{X_{l^{'},1,A'}^{s^{'}, p^{'}}}{\sum_{p'} \sum_{s'} X_{l^{'},1,A^{'}}^{s^{'},p^{'}}} \right)Z_{l,k,p,s^{'},l^{'},1,p^{'}} \right]$$

(7)

##### Probability of transmission per partnership

The probability of transmission per partnership depends on (i) the probability of transmission per sex act, and (ii) the number of sex acts during the partnership (which depends on the risk group of each partner). The probability of transmission per sex act depends on an individual’s circumcision status (if male), in addition to their partner’s state of HIV infection (including ART use), circumcision status (if male), and the degree of condom use in the partnership (which depends on the risk group of each partner).

A baseline transmission probability from uncircumcised males to females is assumed (β_0_). The difference in acquisition and transmission per sex act for other factors (e.g. stage of infection) is specified with respect to this baseline transmission probability using a multiplicative factor. The probability of HIV transmission per sex act is given by $\beta_{k}^{s^{'}k^{'}}$and depends on: s’ (partner’s HIV status), k’ (partner’s circumcision status (if male)), and k (individual’s circumcision status (if male)). The probability of transmission from males to females is assumed to be identical to that for transmission from females to males. Male circumcision is assumed to reduce the risk of acquisition but not onward transmission.

The number of sex acts in a partnership depends on the risk group of both partners and is given by the matrix *n_sex_(l,l’)*. Condom use is modeled as a proportion of sex acts in which condoms are used via the matrix *CU(l,l’)*, which defines condom use in a partnership between an individual’s risk group l and their partner’s risk group l’, modulated by any increase in condom use due to changes over time $\bar{q}$(t). The efficacy of condoms is given as *ϖ*.

The probability of transmission per partnership $Z_{l,k,p,s^{'},l^{'},k^{'},p^{'}}$ is defined as:

For women and for uncircumcised men:

$$Z_{l,k,1,s^{'},l^{'},1,p^{'}}=1-\left( \left( 1-\beta_{1,k}^{s^{'}k^{'}}{\varpi)}^{X} \right)\left( (1-\beta_{1,k}^{s^{'}k^{'}})^{\bar{X}} \right. \right)$$

For circumcised men:

$$Z_{l,3,1,s^{'},l^{'},k^{'},p^{'}}=1-\left( \left( 1-\beta_{1,3}^{s^{'}k^{'}}{\varpi)}^{X} \right)\left( (1-\beta_{1,3}^{s^{'}k^{'}})^{\bar{X}} \right. \right)$$

(8)

Where:

$$X=CU(l,l^{'})\bar{q}_{(t)}n_{sex}\left( l,l^{'} \right)$$

$$\bar{X}=\left( 1-CU(l,l^{'})\bar{q}_{\left( t \right)} \right)n_{sex}\left( l,l^{'} \right)$$

(9)

That is, *X* is the number of sex acts protected by condoms in a partnership between an individual of risk group *l* and their partner of risk group *l’* and $\bar{X}$ is the number of sex acts not protected by condoms in a partnership between an individual of risk group *l* and their partner of risk group *l’*.

##### Sexual mixing

The mixing pattern is defined with respect to sex, five-year age group and behavioural risk group. The proportion of sexual partnerships that an individual of sex g (where g=1 refers to females and g=2 to males), 5 year age group A and risk group *l* forms with an individual of the opposite sex, age group A*’* and risk group *l’*, is given by $\rho_{g,A,l,A^{'}l^{'}}$, and is defined as:

$$P_{1,A,l,A^{'}l^{'}}=\varepsilon_{A}\varepsilon_{l}\left( \delta_{A,A^{'}}\delta_{l,l^{'}} \right)+\left( 1-\varepsilon_{A} \right)\varepsilon_{l}\left( \delta_{l,l^{'}}\frac{C_{2,A^{'},l^{'}}\sum_{k^{'}=2}^{3} \sum_{p^{'}} \sum_{s^{'}} X_{l^{'},k^{'},A^{'}}^{s^{'},p^{'}}}{\sum_{A^{'}} C_{2,A^{'},l^{'}}\sum_{k^{'}=2}^{3} \sum_{p^{'}} \sum_{s^{'}} X_{l^{'},k^{'},A^{'}}^{s^{'},p^{'}}} \right)+\varepsilon_{A}\left( 1-\varepsilon_{l} \right)\left( \delta_{A,A^{'}}\frac{C_{2,A^{'},l^{'}}N_{g^{'}}\left( A^{'},l^{'} \right)}{\sum_{l^{'}} C_{2,A^{'},l^{'}}\sum_{k^{'}=2}^{3} \sum_{p^{'}} \sum_{s^{'}} X_{l^{'},k^{'},A^{'}}^{s^{'},p^{'}}} \right)+\left( 1-\varepsilon_{A} \right)\left( 1-\varepsilon_{l} \right)\left( \frac{C_{2,A^{'},l^{'}}N_{g^{'}}\left( A^{'},l^{'} \right)}{\sum_{A^{'}} \sum_{l^{'}} C_{2,A^{'},l^{'}}\sum_{k^{'}=2}^{3} \sum_{p^{'}} \sum_{s^{'}} X_{l^{'},k^{'},A^{'}}^{s^{'},p^{'}}} \right)$$

$$P_{2,A,l,A^{'}l^{'}}=\varepsilon_{A}\varepsilon_{l}\left( \delta_{A,A^{'}}\delta_{l,l^{'}} \right)+\left( 1-\varepsilon_{A} \right)\varepsilon_{l}\left( \delta_{l,l^{'}}\frac{C_{1,A^{'},l^{'}}\sum_{p^{'}} \sum_{s^{'}} X_{l^{'},1,A^{'}}^{s^{'},p^{'}}}{\sum_{A^{'}} C_{1,A^{'},l^{'}}\sum_{p^{'}} \sum_{s^{'}} X_{l^{'},1,A^{'}}^{s^{'},p^{'}}} \right)+\varepsilon_{A}\left( 1-\varepsilon_{l} \right)\left( \delta_{A,A^{'}}\frac{C_{1,A^{'},l^{'}}\sum_{p^{'}} \sum_{s^{'}} X_{l^{'},1,A^{'}}^{s^{'},p^{'}}}{\sum_{l^{'}} C_{1,A^{'},l^{'}}\sum_{p^{'}} \sum_{s^{'}} X_{l^{'},1,A^{'}}^{s^{'},p^{'}}} \right)+\left( 1-\varepsilon_{A} \right)\left( 1-\varepsilon_{l} \right)\left( \frac{C_{1,A^{'},l^{'}}\sum_{p^{'}} \sum_{s^{'}} X_{l^{'},1,A^{'}}^{s^{'},p^{'}}}{\sum_{A^{'}} \sum_{l^{'}} C_{1,A^{'},l^{'}}\sum_{p^{'}} \sum_{s^{'}} X_{l^{'},1,A^{'}}^{s^{'},p^{'}}} \right)$$

Note: $\sum_{A^{'}} \sum_{l^{'}} \rho_{g,A,l,A^{'},l^{'}}=1$

(10)

The parameter *C_g,A,l_* gives the mean number of partners in a year per individual of sex *g* in age group A and risk group *l*. The degree of assortativity in mixing with respect to age and with respect to risk group are given by ε_A_ and ε_l_, respectively. The identity matrix with respect to risk is given by *δ_l,l’_* whereby:

$$\delta_{l,l^{'}}=\left\{ \begin{aligned} 1, if l=l^{'} \\ 0, if l\neq l^{'} \end{aligned} \right.$$

(11)

A discrepancy matrix $D_{A_{2},l_{2},A_{1},l_{1}}$is defined to balance the number of sexual partnerships between males and females formed with respect to each age group and risk group, where *A_2_* and *l_2_* are the age and risk group of the male partner and *A_1_* and *l_1_* are the age and risk group of the female partner. It is calculated as follows:

$$D_{A_{2},l_{2},A_{1},l_{1}}= \frac{\rho_{2,A,l,A^{'}l^{'}}C_{2,A,l}\sum_{k=2}^{3} \sum_{p} \sum_{s} X_{l,k,A}^{s,p}}{\rho_{1,A,l,A^{'}l^{'}}C_{1,A,l}\sum_{p} \sum_{s} X_{l,1,A}^{s,p}}$$

(12)

The extent to which balancing of the number of sexual partnerships is male-driven is determined by parameter *θ*. When *θ=0.5* the sexes compromise equally. Balancing the number of sexual partnerships is carried out with respect to both partners’ age and risk groups and is represented by:

$$\rho_{2,A,l,A^{'}l^{'}}\longrightarrow D_{A_{2},l_{2},A_{1},l_{1}}{}^{(\theta-1)}{\rho_{2,A,l,A^{'}l^{'}}}$$

$$\rho_{1,A,l,A^{'}l^{'}}\longrightarrow D_{A_{2},l_{2},A_{1},l_{1}}{}^{(\theta)}{\rho_{1,A,l,A^{'}l^{'}}}$$

(13)

| **Parameter** | **Symbol** | **Value** | **Notes** |
| --- | --- | --- | --- |
| Fraction of women in “low risk” group | *ψf(1)* | 0.25 | Calibrated |
| Mean sexual contact rate low | *Cm(1)* | 1.52 year^-1^ | Calibrated |
| Fraction of women in “medium risk” group | *ψf(2)* | 0.48 |  |
| Mean sexual contact rate “medium risk” women | *Cf(2)* | 1.68 year^-1^ | Calibrated |
| Fraction of women in “high risk” group | *ψf(3)* | 0.27 | Calibrated |
| Mean sexual contact rate “high risk” women | *Cf(3)* | 69.1 year^-1^ | Calibrated |
| Fraction of men in “low risk” group | *ψm(1)* | 0.02 |  |
| Mean sexual contact rate “low risk” men | *Cm(1)* | 4.3 year^-1^ | Calibrated |
| Fraction of men in “medium risk” group | *ψm(2)* | 0.69 | Note all remaining men are assumed to be “high risk” |
| Mean sexual contact rate “medium risk” men | *Cm(2)* | 0.55 year^-1^ | Calibrated |
| Fraction of men in the “high risk” group | *ψm(3)* | 0.29 | Calibrated |
| Mean sexual contact rate “high risk” men | *Cm(3)* | 32.6 year^-1^ | Calibrated |

Table S2: Behavioural parameters and values

The number of sex acts per partnership depends on behavioural risk group. The “low risk” groups are intended to reflect long-term stable partnerships and these are assumed to have a high number of sex acts overall. A value of 100 sex acts each year is assumed based on reported frequency of sex in marital relationships in Southern Africa [12].Those in the higher risk groups tend to form more partnerships, but each of these partnerships comprises fewer sex acts and higher condom use. A value of two sex acts is assumed as a representative assumption of casual and commercial sex.

| **Parameter** | **Symbol** | **Value** | **Source** |
| --- | --- | --- | --- |
| Baseline transmission probability from uncircumcised males in the asymptomatic stage of HIV infection to females in a single act of unprotected sex | β_0_ | 0.00157 | Calibrated. The parameter is representative and captures impact of other risk factors not explicitly models such as infection with STIs other than HIV [13,14]. |
| **Factor increase in transmission:** |  |  |  |
| From population with acute HIV infection | $\beta_{p,k}^{1,k^{'}}$ | 27 | [6] |
| From population with chronic HIV infection and CD4 >350 cells/μL | $\beta_{p,k}^{2,k^{'}}$ | 1 | The baseline transmission probability is assumed to apply from the end of acute infection until the period of heightened infectiousness 19-10 months before death [6]. |
| From population with chronic HIV infection and CD4 >200 cells/μL but <350 cells/μL | $\beta_{p,k}^{3,k^{'}}$ | 1.6 |  |
| From population in late infection | $\beta_{p,k}^{4,k^{'}}$ | 3.8 | [6] |
| From population in pre-AIDS | $\beta_{p,k}^{5,k^{'}}$ | 3.8 |  |
| From population in AIDS | $\beta_{p,k}^{6,k^{'}}$ | 3.8 |  |
| From population on early ART | $\beta_{p,k}^{8,k^{'}}$ | 0.08 | [7] |
| From population on late ART | $\beta_{p,k}^{10,k^{'}}$ | 0.08 | [7] |
| From population who have dropped out of ART | $\beta_{p,k}^{11,k^{'}}$ | 3.56 | Estimated |
| From women | $\beta_{p,k}^{s^{'},1}$ | 1 | Transmission from males to females is assumed to be the same as that from females to males. |
| From uncircumcised men | $\beta_{p,k}^{s^{'},2}$ | 1 |  |
| From circumcised men | $\beta_{p,k}^{s^{'},3}$ | 1 | Assumes no effect of circumcision on  HIV transmission. |
| To circumcised men | $\beta_{p,3}^{s^{'},k^{1}}$ | 0.4 | Risk of HIV acquisition is 60% lower than among uncircumcised men [15–17] |
| Condom efficacy | ☐ | 0.1 | Assumes condoms provide 90% protection from HIV infection |

**Table S3: Factor increments in transmission probability per sex act with respect to baseline transmission probability (β_0_)**

##### Force of infection coefficient

To model the introduction of interventions from 2019 until 2050, a coefficient acts on the force of infection equally across all age and behavioural risk groups. The coefficient is time-dependent and represents the scale up of condom usage, oral pre-exposure prophylaxis (PrEP), long-acting injectable PrEP (LA PrEP), and a vaccine. The rate of scale-up of each of these interventions is constant during the scale-up period. The coefficient is calculated in the equation below:

$${F_{t} = [Cov}_{PrEP, t} {(1- Eff}_{PrEP})]\times{[Cov}_{LA PrEP, t} {(1- Eff}_{v})]\ldots\times{[Cov}_{condoms, t} {(1- Eff}_{condoms})]\times{[Cov}_{vaccine,t} {(1- Eff}_{vaccine})]$$

(14)

where *F_t_* is the coefficient of the force of infection at time *t*, ${Cov}_{intervention, t}$represents the coverage of a particular intervention at time *t* and ${Eff}_{intervention}$ represents its efficacy. The scale-up periods and coverage changes are given in Table 1 (main manuscript).

# 2.5 Male circumcision

The level of circumcision changes over time to reflect the increase in male circumcision as has occurred in recent years according to a nationally representative survey and is projected to continue increasing in the future [11] (Figure S2) . The movement of

uncircumcised men to circumcised classes is represent by the function $g\left( k \right)$, included in the equations below.

$$g\left( 1 \right)=0$$

$$g\left( 2 \right)= \frac{dX_{l,2,a}^{0,p}}{dt}-\eta_{C}$$

$$g\left( 3 \right)= \frac{dX_{l,3,a}^{0,p}}{dt}+\eta_{C}$$

(15)

The parameter $\eta_{C}$ gives the scale-up rate for male circumcision, which is a time-varying parameter based on the extent to which the current level of circumcision in the sexually active adult population matches the data on circumcision prevalence. Movement from uncircumcised to circumcised classes occurs at age 15 to represent circumcision that has occurred after birth but before entering the sexually active population. In the model, the rate of HIV acquisition for circumcised men is reduced by 60% [15–17].


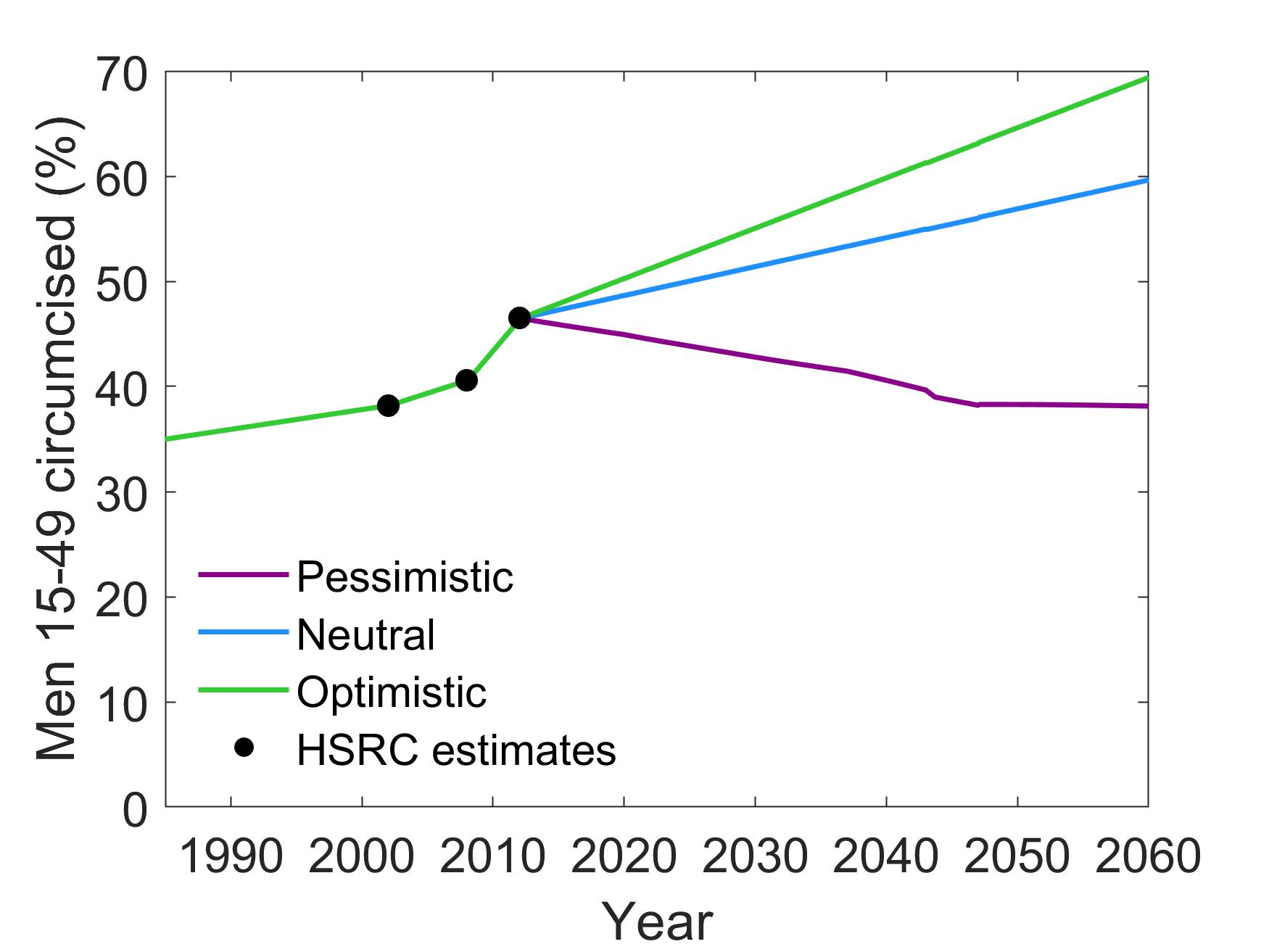


***Figure S2. The proportion of adult men that are circumcised with respect to time.***

*The level of circumcision in the model was calibrated to data reported in a nationally representative survey* [11]*. Future circumcision trends follow three trajectories according to the Pessimistic Neutral and Optimistic scenarios.*

# 2.6 Antiretroviral Treatment

ART can be initiated for the population with four programme types, specified with different initiation rules, as described above. A drop-out rate of 0.02 is assumed, regardless of the CD4 level at which ART is initiated. A rate of progressing to AIDS of 0.105 is assumed for those initiating ART below 200 cells/μL. The survival probability (p) of 0.9 reported by Mahy et. al. [18] was converted to a per capita mortality rate (r), using: p =1 - e^-rt^ . A rate of progressing to AIDS of 0.013 is assumed for those initiating ART above 200 cells/μL. A crude death rate of 1.3 deaths per 100 person years had been reported among individuals receiving early ART in the USA and Canada [19]. The number of individuals receiving ART is calibrated to the total number of people on ART in South Africa [20,21] (Figure S3).


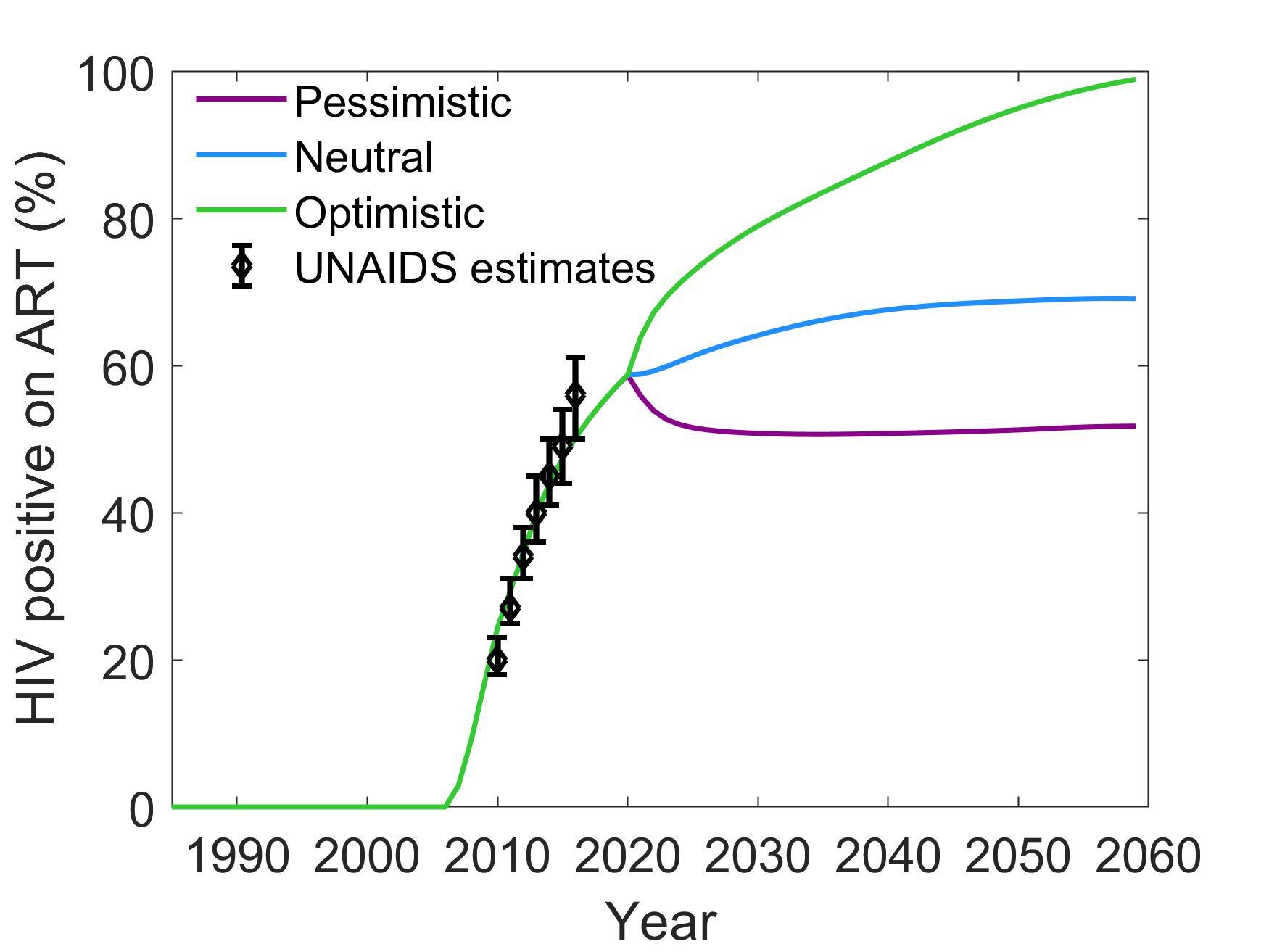


***Figure S3. The percentage of HIV positive adults (15-49) receiving antiretroviral therapy in South Africa.***

*Model data is compared to estimates of the percentage of HIV positive adults on ART in South Africa* [20,21]*. Future trends in ART vary according to the Pessimistic, Neutral and Optimistic scenarios.*

# 2.7 Contraception

We model the use of different contraceptives by splitting the female population into seven contraceptive compartments: no method, oral hormonal contraceptives, DMPA, norethisterone enanthate (NET-EN), copper intrauterine device (IUD), female sterilisation and ‘other’ methods.

| **Contraceptive** | **Efficacy (typical use)** | **One year discontinuation rates (%)** | **Source** |
| --- | --- | --- | --- |
| No method | 15% |  | [22] |
| Combined oral contraceptive | 91% | 33 | [22] |
| DMPA | 94% | 44 | [22] |
| NET-EN | 94% | 44 | Assumed to be the same as DMPA |
| Copper IUD | 99.2% | 22 | [22] |
| Female sterilisation | 99.5% |  | [22] |
| Other methods | 83.9% | 33 (assumed to be the same as oral contraceptives) | average of the efficacies of: male sterilisation, withdrawal, fertility awareness and male condoms [22] |

***Table S4. Contraceptive efficacy and continuation rates for methods used in the model.***

Condom use is modelled separately to female-controlled contraceptives; it is used to impact upon the rate of HIV transmission (section 2.4).

The equations describing movement between contraceptive classes are given below.

$$q\left( p \right)= {{X_{l,k,a}^{s,p}}_{15-49}}_{(t+1)}- {{X_{l,k,a}^{s,p}}_{15-49}}_{(t)}$$

For *p* = 2:7

${{X_{l,k,a}^{s,p}}_{15-49}}_{(t+1)}= {{X_{l,k,a}^{s,p}}_{15-49}}_{(t)}+ \eta_{p}{{X_{l,k,a}^{s,1}}_{15-49}}_{(t)}- \sigma_{p}{{X_{l,k,a}^{s,p}}_{15-49}}_{(t)}$

For *p* = 1 (no method)

${{X_{l,k,a}^{s,1}}_{15-49}}_{(t+1)}= {{X_{l,k,a}^{s,1}}_{15-49}}_{(t)}+ \sum_{p=2}^{7} \sigma_{p}{{X_{l,k,a}^{s,p}}_{15-49}}_{(t)}- \sum_{p=2}^{7} \eta_{p}{{X_{l,k,a}^{s,1}}_{15-49}}_{(t)}$

(16)

Women move away from contraceptives to the “no method” compartment based on the discontinuation rates ( $\sigma_{p}$ , Table S4). The rate of uptake $\eta_{p}$ of each method is calibrated such that contraceptive prevalences approximate those reported in the South African National HIV Prevalence, Incidence and Behaviour Survey, 2012 [11].

# 2.8 Further Model calibration

The behavioural parameters and the baseline transmission probability were calibrated as these are difficult to empirically estimate reliably. Rates of contraceptive uptake were calibrated to fit contraceptive data. To account for the decrease in average fertility due to the addition of contraceptive classes, the baseline fertility rate was increased and calibrated using population size data. Calibrations are shown in supplementary Figures S2, S3, S4 and S5.


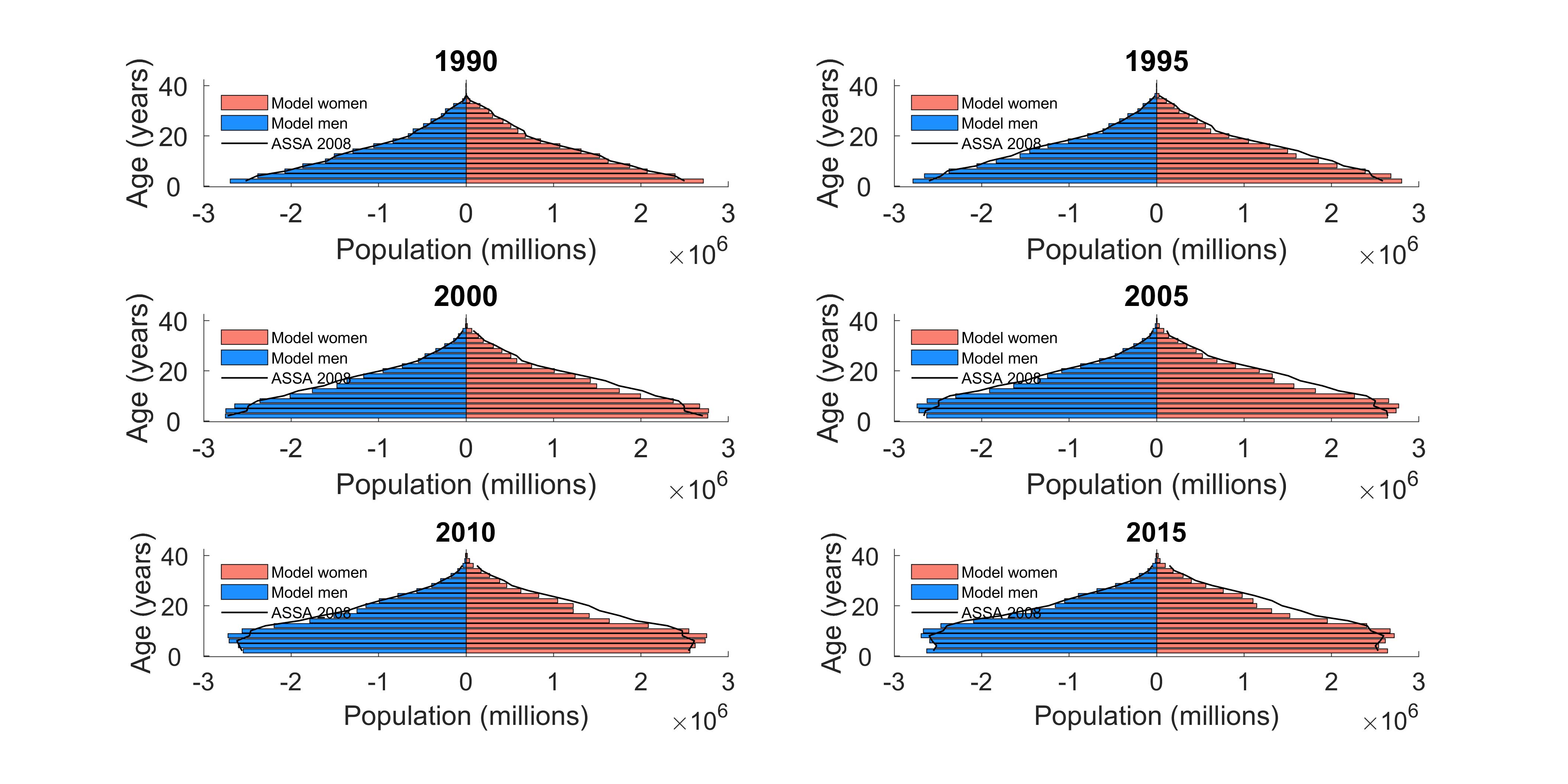


***Figure S4. Population pyramids for South Africa for 1985, 1990, 1995, 2000, 2005 and 2010.*** *Model population structure is compared to annual age-structured population size model estimates produced by the Actuarial Society of South Africa* [10]*.*


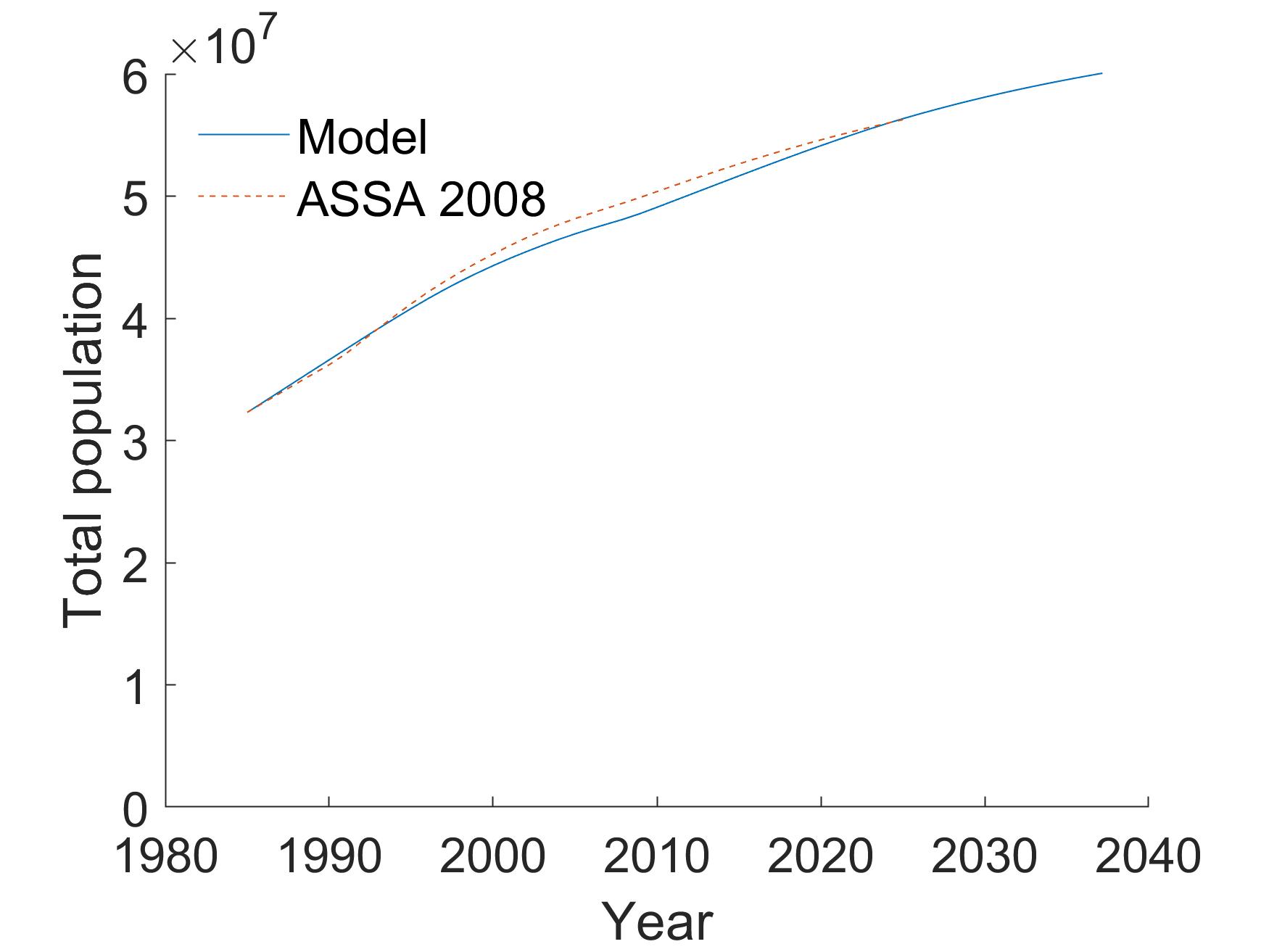


***Figure S5. Population size with respect to time.***

*The total population of the model was calibrated to previous estimates from a demographic model of the South African population* [10]*.*

*
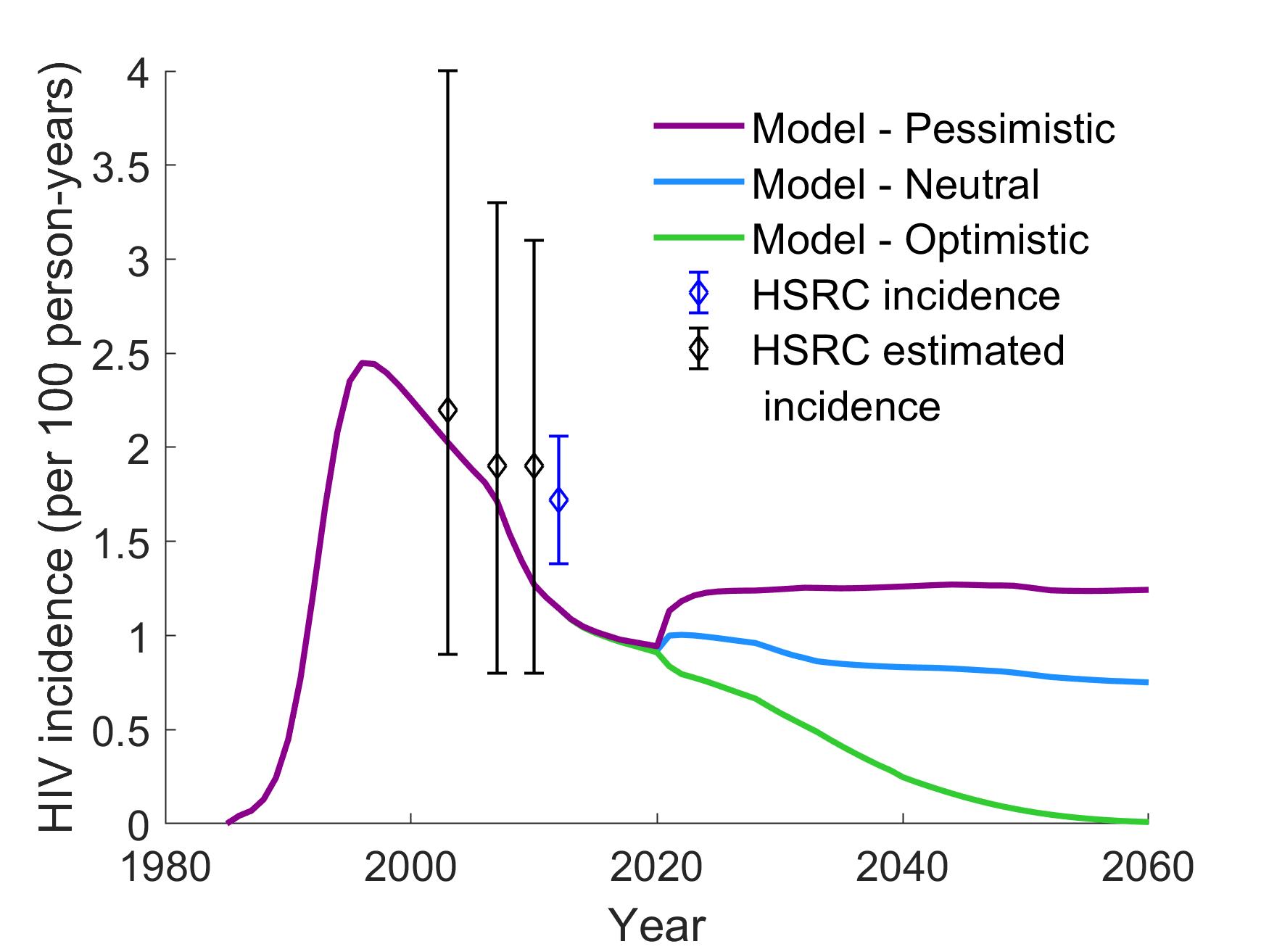
*

***Figure S6 HIV incidence in 15-49 year olds***

*HIV incidence in adults was calibrated to incidence data from a nationally representative survey as well as incidence estimates produced by a mathematical model calibrated to prevalence data* [11]*. Model incidence is shown for the Pessimistic, Neutral and Optimistic scenarios.*

*
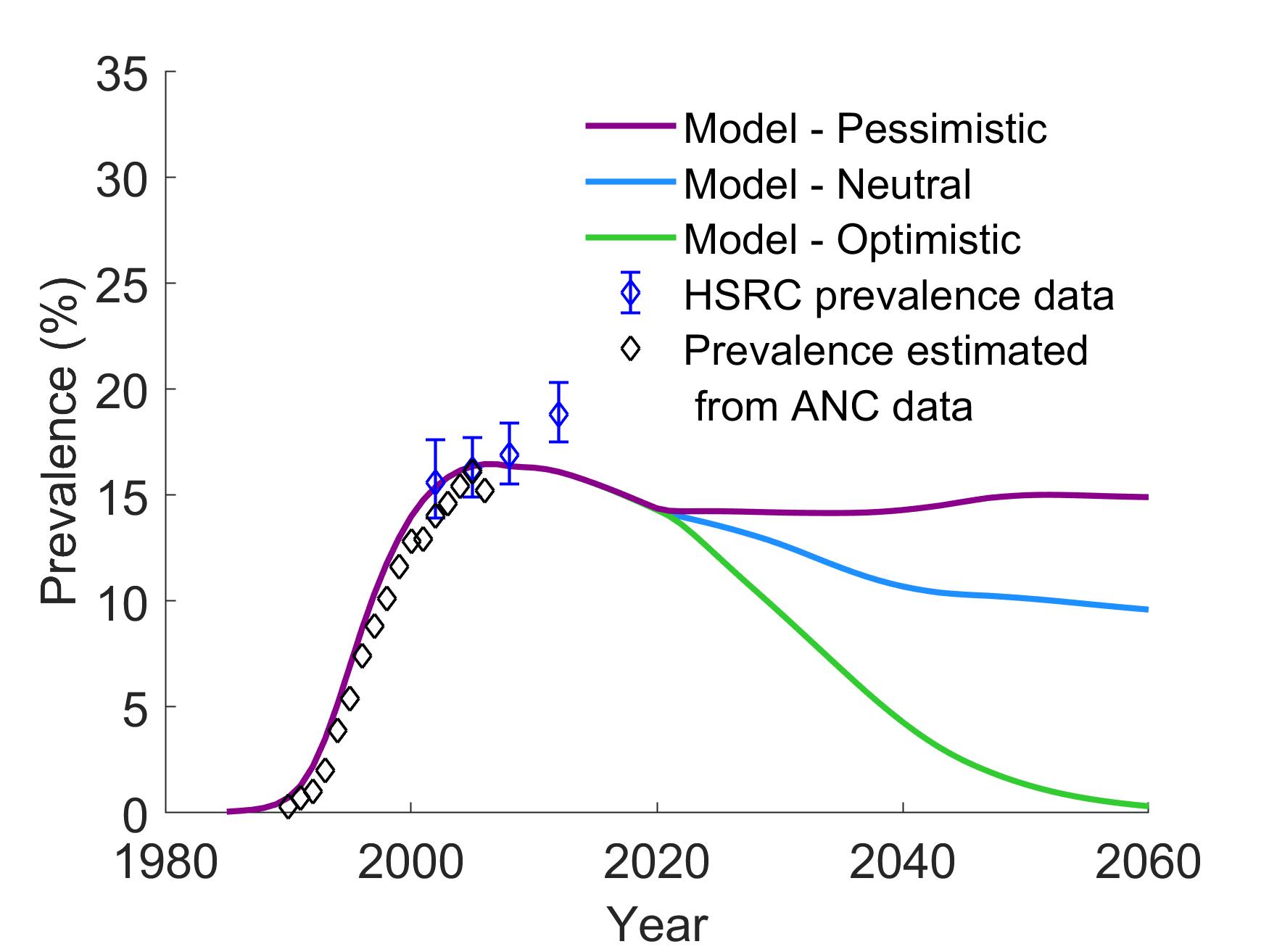
*

***Figure S7. Prevalence in 15-49 year olds.***

*Adult HIV prevalence is calibrated to nationally representative survey data from South Africa as well as UNAIDS prevalence estimates* [11,21,24]*. Model prevalence is shown for the Pessimistic, Neutral and Optimistic scenarios.*

*
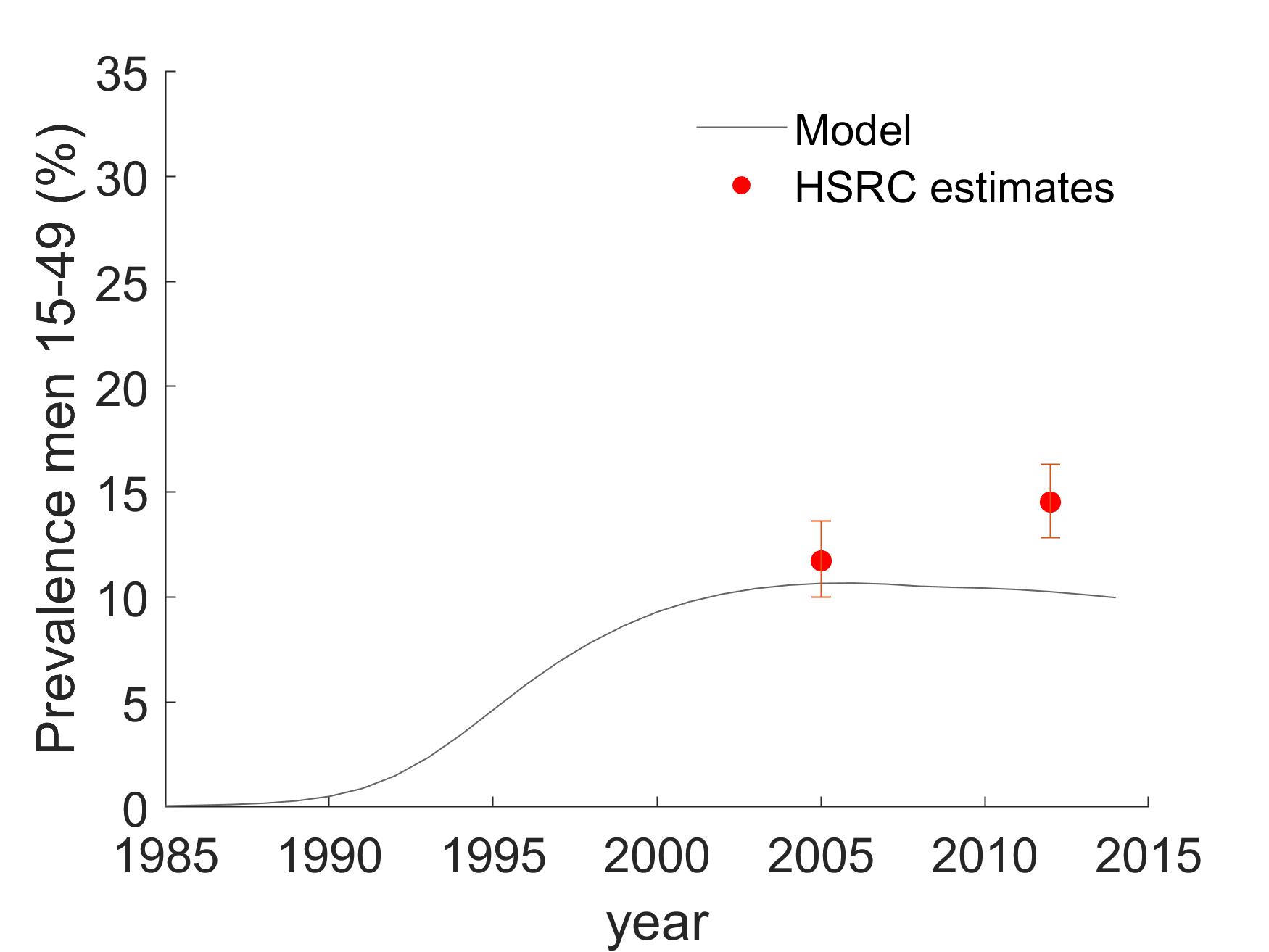
*

***Figure S8. Male HIV prevalence***

*HIV prevalence in the model was calibrated to sex-specific prevalence data* [11]*.*

***
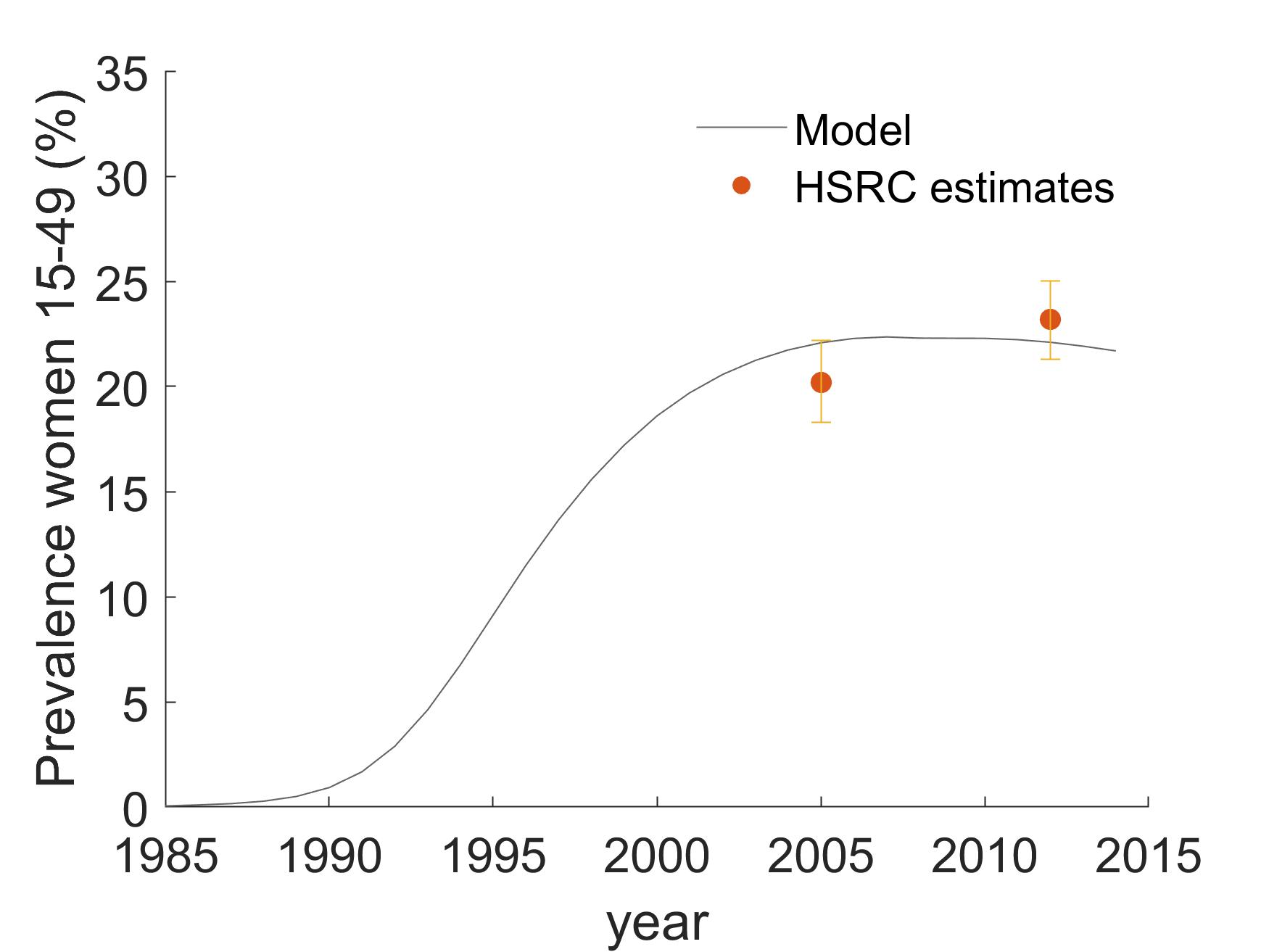
***

***Figure S9. Female HIV prevalence***

*HIV prevalence in the model was calibrated to sex-specific prevalence estimates* [11] *.*

***
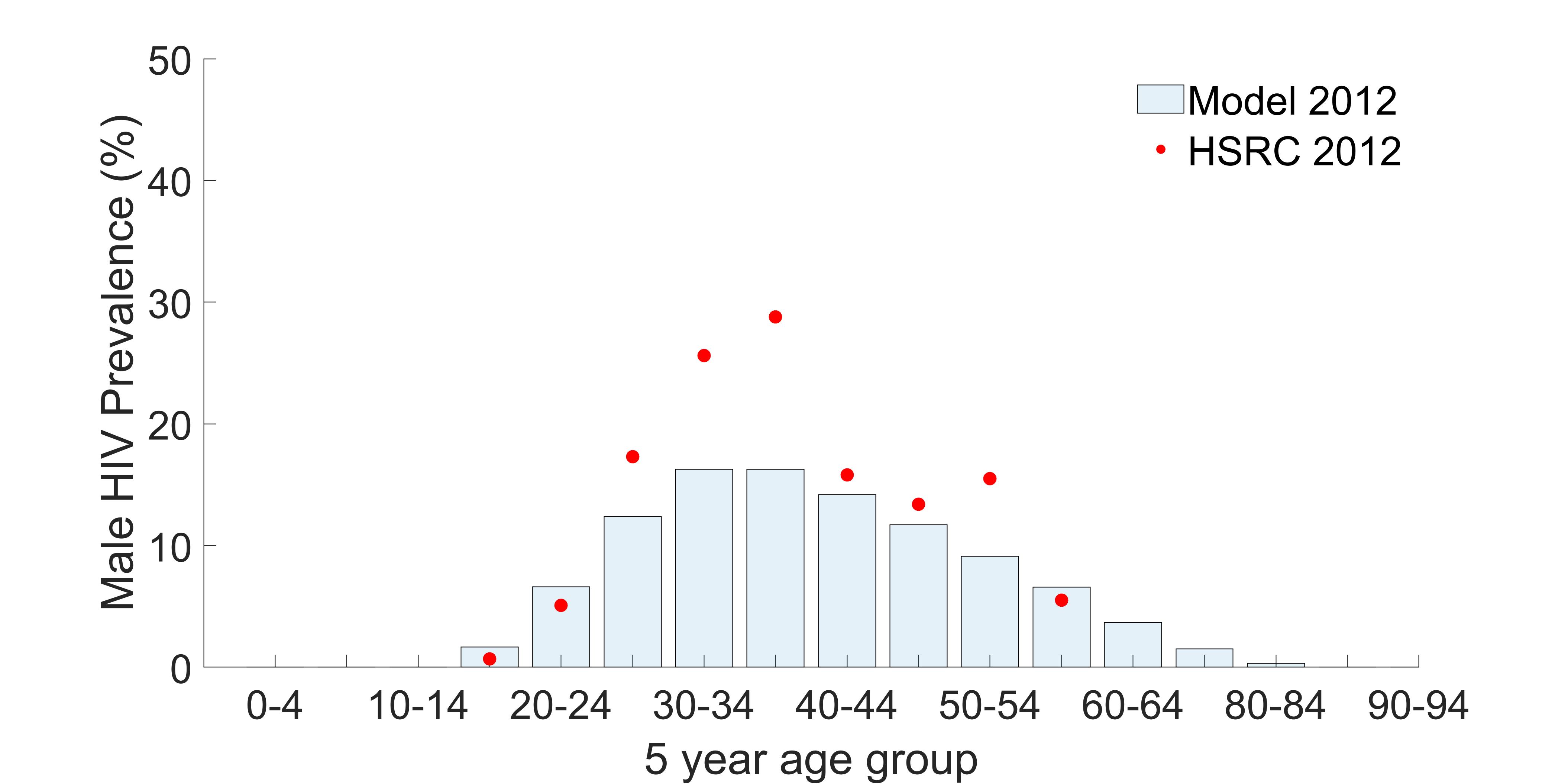
***

***Figure S10. Male age-specific HIV prevalence***

*HIV prevalence in the model was calibrated to male age-specific prevalence estimates* [11]*.*

***
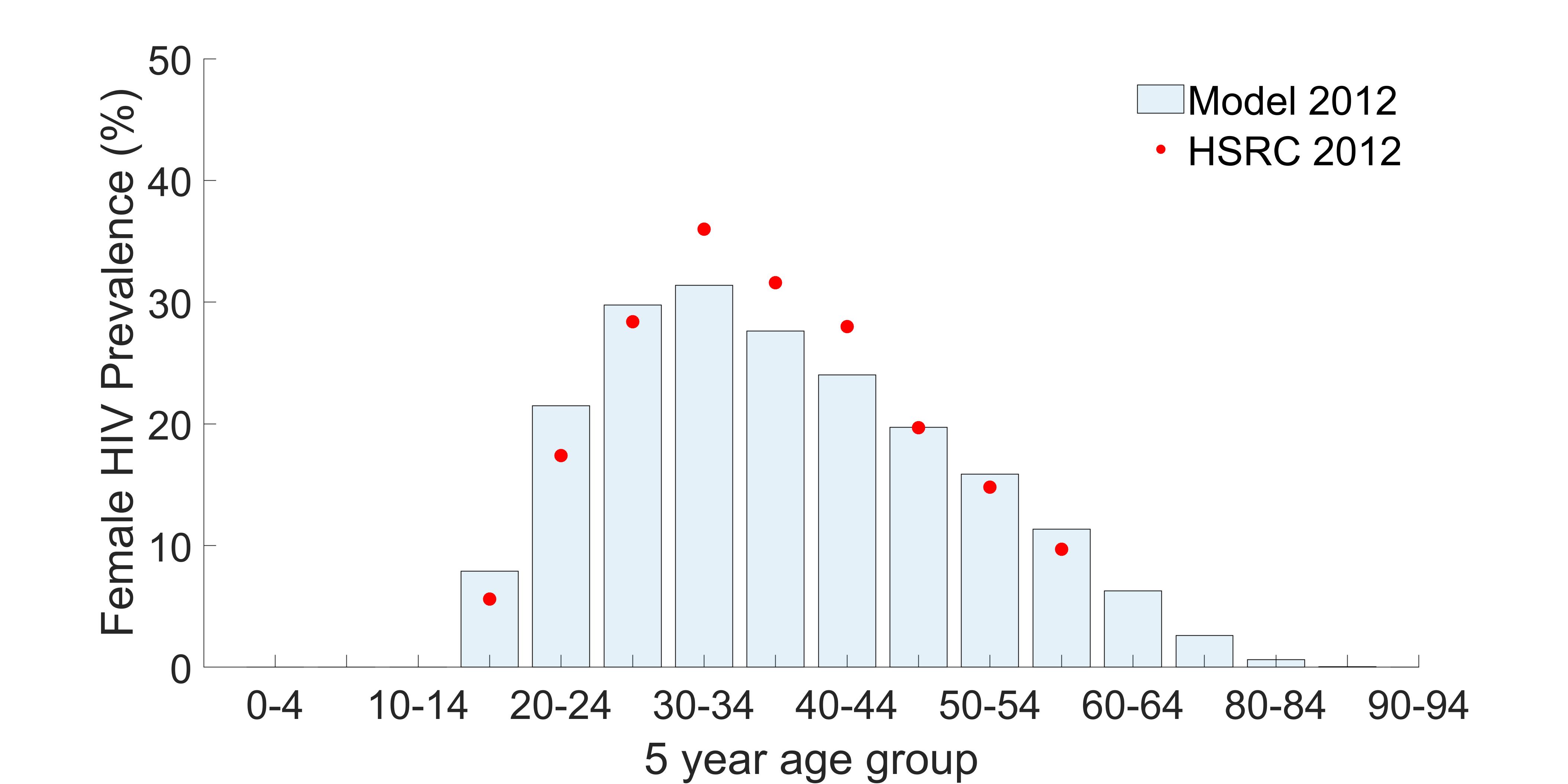
***

***Figure S11. Female age-specific HIV prevalence***

*HIV prevalence in the model was calibrated to female age-specific prevalence estimates* [11] *.*

# References

1 Cremin I, Alsallaq R, Dybul M, Piot P, Garnett G, Hallett TB. **The new role of antiretrovirals in combination HIV prevention: a mathematical modelling analysis**. *Aids* 2013; **27**:447–458.

2 Smith JA, Anderson SJ, Harris KL, McGillen JB, Lee E, Garnett GP, *et al.* **Maximising HIV prevention by balancing the opportunities of today with the promises of tomorrow: a modelling study**. *Lancet HIV* 2016; **3**:e289–e296.

3 Anderson RM, May RM. *Infectious diseases of humans: dynamics and control*. Oxford: Oxford University Press; 1991.

4 Garnett GP, Anderson RM. **Factors controlling the spread of HIV in heterosexual communities in developing countries: patterns of mixing between different age and sexual activity classes**. *Philos Trans R Soc London B Biol Sci* 1993; **342**.http://rstb.royalsocietypublishing.org/content/342/1300/137.long (accessed 23 May2017).

5 Garnett GP, Anderson RM. **Sexually transmitted diseases and sexual behavior: insights from mathematical models.** *J Infect Dis* 1996; :S150-61.

6 Hollingsworth TD, Anderson RM, Fraser C. **HIV-1 transmission, by stage of infection.** *J Infect Dis* 2008; **198**:687–93.

7 Donnell D, Baeten JM, Kiarie J, Thomas KK, Stevens W, Cohen CR, *et al.* **Heterosexual HIV-1 transmission after initiation of antiretroviral therapy: a prospective cohort analysis.** *Lancet* 2010; **375**:2092–8.

8 Cohen MS, Chen YQ, McCauley M, Gamble T, Hosseinipour MC, Kumarasamy N. **Prevention of HIV-1 infection with early antiretroviral therapy**. *N Engl J Med* 2011; **365**:493–505.

9 Lodi S, Phillips A, Touloumi G, Geskus R, Meyer L, Thiébaut R, *et al.* **Time from human immunodeficiency virus seroconversion to reaching CD4+ cell count thresholds <200, <350, and <500 Cells/mm3: Assessment of need following changes in treatment guidelines**. *Clin Infect Dis* 2011; **53**:817–825.

10 Africa AS of S. **ASSA 2008 Model**. 2011.

11 Shisana O, Rhele T, Simbayi LC, Zuma K, Jooste S, Zungu N, *et al.* *South African National HIV Prevalence, Incidence and Behaviour Survey, 2012*. Cape Town: HSRC Press; 2012.

12 BROWN MS. **Coitus, the proximate determinant of conception: inter-country variance in sub-Saharan Africa.** *J Biosoc Sci* 2000; **32**:145–159.

13 Boily M-C, Baggaley RF, Wang L, Masse B, White RG, Hayes RJ, *et al.* **Heterosexual risk of HIV-1 infection per sexual act: systematic review and meta-analysis of observational studies.** *Lancet Infect Dis* 2009; **9**:118–29.

14 Wawer MJ, Gray RH, Sewankambo NK, Serwadda D, Li X, Laeyendecker O, *et al.* **Rates of HIV-1 transmission per coital act, by stage of HIV-1 infection, in Rakai, Uganda.** *J Infect Dis* 2005; **191**:1403–9.

15 Bailey RC1, Moses S, Parker CB, Agot K, Maclean I, Krieger JN, Williams CF, Campbell RT N-AJ. **Male circumcision for HIV prevention in young men in Kisumu, Kenya: a randomised controlled trial.** *Lancet* 2007; **369**:643–656.

16 Auvert B, Taljaard D, Lagarde E, Sobngwi-Tambekou J, Sitta R, Puren A. **Randomized, controlled intervention trial of male circumcision for reduction of HIV infection risk: The ANRS 1265 trial**. *PLoS Med* 2005; **2**:1112–1122.

17 Gray RHM, Kigozi G, Serwadda D, Makumbi F, Watya S, Nalugoda F, *et al.* **Male circumcision for HIV prevention in men in Rakai, Uganda: a randomised trial**. *Lancet* 2007; **369**:657–666.

18 Mahy M, Lewden C, Brinkhof MWG, Dabis F, Tassie J-M, Souteyrand Y, *et al.* **Derivation of parameters used in Spectrum for eligibility for antiretroviral therapy and survival on antiretroviral therapy**. *Sex Transm Infect* 2010; **86**:ii28-ii34.

19 Kitahata MM, Gange SJ, Abraham AG EA. **Effect of early versus deferred antiretroviral therapy for HIV on survival.** *N Engl J Med* 2009; **360**:1815–1826.

20 Johnson, Francis L. **Access to antiretroviral treatment in South Africa, 2004 - 2011**. *South Afr J HIV Med* 2012; **13**:22–27.

21 UNAIDS. **AIDSInfo**. 2014.http://aidsinfo.unaids.org/ (accessed 6 Jun2016).

22 Trussell J. **Contraceptive Efficacy**. In: *Contraceptive Technology*.New York: Ardent Media; 2011. pp. 779–863.

23 National Department of Health (NDoH), Statistics South Africa (Stats SA) SAMR, Council (SAMRC) and I. *South Africa Demographic and Health Survey 2016: Key Indicators*. Pretoria, South Africa, and Rockville, Maryland, USA: NDoH, Stats SA, SAMRC, and ICF; 2017.

24 Granich RM, Gilks CF, Dye C, De Cock KM, Williams BG. **Universal voluntary HIV testing with immediate antiretroviral therapy as a strategy for elimination of HIV transmission: a mathematical model**. *Lancet* 2009; **373**:48–57.
